# Efficient Convex Optimization for Energy-Based Acoustic Sensor Self-Localization and Source Localization in Sensor Networks

**DOI:** 10.3390/s18051646

**Published:** 2018-05-21

**Authors:** Yongsheng Yan, Haiyan Wang, Xiaohong Shen, Bing Leng, Shuangquan Li

**Affiliations:** 1 Centre for Infocomm Technology (INFINITUS), School of Electrical and Electronic Engineering, Nanyang Technological University, 50 Nanyang Avenue, Singapore 639798 , Singapore; 2 Key Laboratory of Ocean Acoustics and Sensing, Ministry of Industry and Information Technology, Xi’an 710072, China; hywang@nwpu.edu.cn (H.W.); xhshen@nwpu.edu.cn (X.S.); 3 School of Marine Science and Technology, Northwestern Polytechnical University, Xi’an 710072, China; leng_bing89@nwpu.edu.cn; 4 Xi’an Institute of Applied Optics, Xi’an 710065, China; lsq850911@163.com

**Keywords:** sensor self-localization, source localization, sensor networks, convex optimization, semidefinite programming

## Abstract

The energy reading has been an efficient and attractive measure for collaborative acoustic source localization in practical application due to its cost saving in both energy and computation capability. The maximum likelihood problems by fusing received acoustic energy readings transmitted from local sensors are derived. Aiming to efficiently solve the nonconvex objective of the optimization problem, we present an approximate estimator of the original problem. Then, a direct norm relaxation and semidefinite relaxation, respectively, are utilized to derive the second-order cone programming, semidefinite programming or mixture of them for both cases of sensor self-location and source localization. Furthermore, by taking the colored energy reading noise into account, several minimax optimization problems are formulated, which are also relaxed via the direct norm relaxation and semidefinite relaxation respectively into convex optimization problems. Performance comparison with the existing acoustic energy-based source localization methods is given, where the results show the validity of our proposed methods.

## 1. Introduction

In recent years, with the advances in distributed and collaborative signal processing and communication, sensor networks have become an attractive system for various civil and military applications, especially in surveillance areas, such as environmental monitoring [1,2], traffic monitoring [3,4], source detection, localization, and tracking [5,6,7,8,9,10], etc. In this paper, we focus on the application of sensor self-localization and source localization. More specifically, the position of sensors or a target is derived by fusing the received acoustic energy from local nodes in a sensor network.

Different measurement models are defined to localize the source, such as time of arrival (TOA) [10,11,12,13], time difference of arrival (TDOA) [14,15,16,17], angle of arrival (AOA) [18], received signal strength (RSS) [19,20,21], received signal energy [5,22,23,24,25,26,27,28,29,30], distance measurement (DM) [31], and a combination of part of them [32,33]. The range information between sensor nodes and the source is reflected in TOA, TDOA, and RSS, while the angular information of the emitting signal relative to self-nodes is reflected in AOA. In comparison, TOA and TDOA based methods require accurate and complicated time synchronization among different sensors or source. The AOA based method requires angle measuring devices (e.g., microphone array). RSS and energy based methods are efficient and cost saving (in both energy and computation capability) in practical application due to its implementation simplicity. In this paper, we focus on the energy-based acoustic source localization method. More specifically, we focused on the solving technique of acoustic energy based source localization, and developed two efficient convex optimization methods based on direct norm relaxation and semidefinite relaxation, respectively.

**Figure 1:**
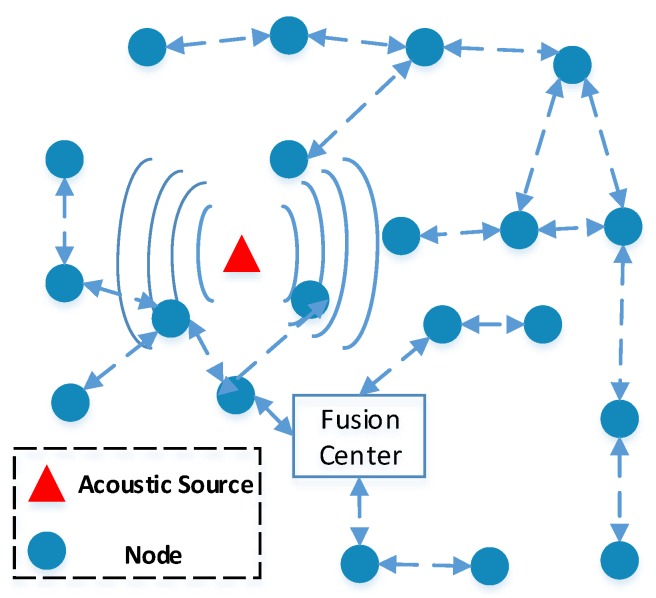
Acoustic nodes (filled circles) receive signal generated by an acoustic source (represented by a filled triangle) and data fusion for source localization.

The scenario of acoustic source localization in a sensor network is depicted in Figure 1. By  considering an acoustic source localization system to learn the migration characteristics of birds, we need to track the position of them. In such a system, several nodes with communication capacity are randomly deployed in a surveillance area, and then form a sensor network. We assume that some nodes have Global Navigation Satellite System (GNSS) modules, which can obtain their own positions via GNSS. Furthermore, the time synchronization by using one pulse per second signal from GNSS satellites is carried out after deployment. If parts of nodes can not receive the GNSS signal, the time synchronization algorithm by using data exchange among unsynchronized nodes and synchronized nodes in [34] can be employed to make sure all the nodes are synchronized. The space initialization is required after time synchronization, which is to obtain the spacial structure of the sensor network. The position of node with GNSS module can be directly derived, while the position of the node without GNSS module can be calculated by using sensor self-localization algorithms based on the given positions of anchor nodes. After the initialization, the sensor network system starts to locate the acoustic target, e.g., the migrated bird. All of these nodes in such a sensor network receive the acoustic signal, which is generated by the acoustic source. The energy reading is calculated according to the measurements by averaging the noisy samples of received signals in each node. Then, the energy reading of each node is transmitted via a wireless channel or cable medium to the fusion center by using time-division multiple access or frequency-division multiple access. The fusing algorithms are our main focus in this paper, which are carried out to efficiently combine the energy readings from all the nodes at a fusion center.

### 1.1. Related Work

Acoustic energy based source localization has been extensively studied in surveillance areas due to its cost saving in both energy and computation capability. In recent years, the first research on acoustic energy-based source localization is traced back to 2003 in [22], where the author proposed an acoustic energy decay model based on the real-field experiment. A quadratic elimination (QE) for the source localization is proposed to derive the location estimate. Following the work of [22], Sheng extended such an energy decay model to the scenario of multiple source localization and proposed a maximum likelihood (ML) method in [5]. The ML estimator has been proved to be asymptotically optimal when the statistic distribution of acoustic energy readings is known. However, it is intractable to derive the solution of the ML estimator due to the severely nonlinear and nonconvex objective function. An iterative method is proposed to solve the ML problem in [23]. As we know, the performance of the iterative method is strongly dependent on the initial value, and an inappropriate initialization may lead to a local optimum or a saddle point of the objective function. Moreover, the computation of solving ML is costly. To reduce the computational complexity of solving ML, the authors in [24] proposed two weighted least-squares (WLS) methods to obtain a good trade-off between performance and complexity. In addition, we presented a direct WLS source localization method in [25], where the source position, the transmission power (or radiation power), together with the quadratic term of source location are simultaneously estimated in a closed-form. All of these WLS based source localization methods have superior performance than the QE method. In order to derive the linear estimation, the LS and WLS based source localization methods cope with the quadratic term with respective to the unknown parameter in different technologies. The core idea is to eliminate the unknown quadratic term. Such a kind of technique leads to higher performance loss under larger noise (or smaller signal-to-noise ratio).

Aiming to efficiently solve the ML problem, and enhance the source localization performance of LS and WLS based methods at higher noise level, some authors in [20,21,26,27,28,29,30] proposed a kind of convex approximation technique to convert the nonconvex optimization problems into convex ones, which can be reliably and efficiently solved to derive globally optimal solution [35,36]. It should be noted that the core idea of acoustic energy based source localization via convex optimization is to convert the original optimization problem into a convex one. Then, the convex problem is easily solved using interior-point methods or other special methods. In [26], by considering the free-space scenario with decay factor 2, a semidefinite relaxation (SDR) technique was employed to derive the convex optimization solution by using the acoustic energy ratio with the reference node. Under the same scenario, an approximate of the original ML problem was proposed by joint estimating (JE) the unknown transmission power and source location in [27]. Based on the same acoustic energy attenuation model with decay factor 2, a quadratic programming problem via SDR (labeled as SQP) was formulated in [29], which requires additional matrix rank-one decomposition to find the optimal source location. However, such a source localization method is quite sensitive to some matrix parameters. As such, the author proposed a combination method that utilizes the advantages of SQP and JE (proposed by Wang in [27]). These source localization methods suffer from the applicable limitation that can only be employed under the free-space energy attenuation. To avoid such a limitation, Wang extended the JE method to the scenario with any energy decay factor in [28]. In addition, by taking logarithmic acoustic energy attenuation into account, the SDR technique was taken full advantage of to transfer the original nonconvex ML optimization problem into convex one in [20,21]. Alternatively, the minimax optimization formulation was developed to directly minimize the mean square error of the unknown parameter estimation error in [30]. All of these convex optimization based source localization methods is based on the Gaussian noise assumption. In this paper, we focus on the scenario with any energy decay factor, which is more practical than the free-space assumption, and propose direct norm relaxation and SDR based source localization methods under maximum likelihood and minimax criterion, respectively. The source localization methods under the minimax criterion are suitable for the non-Gaussian noise assumption.

### 1.2. Contributions

In this paper, we mainly focus on the fusion algorithms to efficiently solve the optimization problem in the application of sensor self-localization and source localization under the acoustic energy attenuation model with any decay factor. More specifically, we focus on the solving technique to derive the source location estimation based on the acoustic energy readings. Several convex optimization problems are derived via a so-called *direct norm relaxation* and SDR, respectively. Our goal here is to address the following questions: (1) how to effectively relax the nonconvex optimization into convex one for the application of the energy-based source localization? (2) how to get the optimal location estimation under colored noises of acoustic energy readings? (3) how does the localization performance vary with different related parameters, such as energy decay factor, sampling number of acoustic signals, source transmission or radiation level, etc.? To answer question (1), a direct norm relaxation and SDR, respectively, are utilized to convert nonconvex problems into convex ones based on the energy attenuation model of [22], which are reliably and efficiently solved in polynomial time. To answer question (2), i.e., to solve the applicable limitation of current convex optimization with colored energy reading noises, a minimax criterion is adopted to develop reduced computational complexity methods. To answer question (3), the localization performance comparison is carried out under different related parameters in Section 6. Our main contributions of this work are summarized as follows.

- For sensor self-localization, a so-called direct norm relaxation and SDR, respectively, are utilized to convert both the nonconvex ML optimization problems and minimax optimization problems into convex ones. These two kinds of relaxations are compared and analyzed.
- For source localization, the ML and minimax optimization formulations based on transmission power-elimination are derived. Based on such formulations, two source localization methods are developed by utilizing the direct norm relaxation and SDR, respectively.
- The Cramér–Rao Low Bound (CRLB) of the energy-based source localization with any energy decay factor for both the sensor self-localization and the source localization is given.

It is noted that this paper is an extension of our work in [26]. The differences with our previous conference paper are given in the following aspects: (1) the energy decay factor can be set as any number, (2) a direct norm relaxation is utilized to relax the nonconvex optimization problem into a convex one, and (3) the performance comparison with the existing acoustic energy based source localization methods is carried out.

The rest of this paper is organized as follows. Section 2 gives the energy decay model. The  maximum likelihood estimation (MLE) and approximate MLE formulations are also derived according to the energy decay model. Section 3 describes the sensor self-localization methods with known transmit power. The source localization with unknown transmission power is discussed in Section 4. The CRLB under any energy decay factor is given in Section 5. The simulation results of the performance comparison are given in Section 6. Finally, Section 7 concludes this paper.

## 2. Problem Statement

### 2.1. System Model

**Figure 2:**
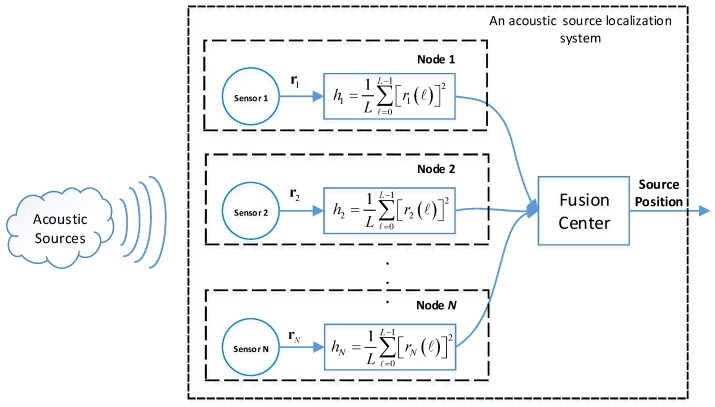
The block digram of energy-based acoustic source localization in a sensor network.

Consider a sensor network shown in Figure 2, which consists of *N* spatially distributed sensors and a fusion center. A source transmits or emits acoustic waves to the surrounding environment. *N* spatially distributed nodes measure the acoustic signal generated by the acoustic source. The received signal at the *n*th sensor is

(1) $$r_{n}\left(\ell \right)=\frac{\sqrt{g_{n}}s\left(\ell /f_{s}-\tau_{n}\right)}{∥x_{n}{-y∥}^{\frac{\alpha }{2}}}+\omega_{n}\left(\ell \right)$$

for the index of nodes N=1,⋯,N and the index of measurements of each node ℓ=1,⋯,L, where $\sqrt{g_{n}}$ is the amplifier gain of the *n*th node, $\tau_{n}$ is the transmission time delay of the acoustic signal from the source to the *n*th node, $s(\ell /f_{s}-\tau_{n})$ is the acoustic intensity measured 1 m away from the source, and $f_{s}$ is the sampling frequency, which is a sampling parameter to sample the continuous signal into a discrete one. $x_{n}$ and y represent the *k*-dimensional (k=2 or k=3) position vector of the *n*th sensor and the source, respectively. $∥x_{n}-y∥$ denotes the Euclidean distance between the *n*th sensor and the source. α is the decay factor depending on the surrounding environment. $\omega_{n}\left(\ell \right)$ is white Gaussian measurement noise with variance $\zeta_{n}^{2}$, i.e., $\omega_{n}\left(\ell \right)∼N\left(0,\zeta_{n}^{2}\right)$. The similar model can be found in [5,24,28].

The received signal energy at sensor *n* can be expressed as

(2) $$\begin{array}{c} E\left[r_{n}^{2}\left(\ell \right)\right]=E\left[\frac{g_{n}s^{2}\left(\ell /f_{s}-\tau_{n}\right)}{∥x_{n}{-y∥}^{\alpha }}\right]+E\left[\frac{2\sqrt{g_{n}}s\left(\ell /f_{s}-\tau_{n}\right)\omega_{n}\left(\ell \right)}{∥x_{n}{-y∥}^{\alpha /2}}\right]+E\left[\omega_{n}^{2}\left(\ell \right)\right], \end{array}$$

where E[·] denotes the expectation of random variable. The cross term $E[\left(s\left(\ell /f_{s}-\tau_{n}\right)\right)\omega_{n}\left(\ell \right)]$ is 0 due to the assumptions of zero-mean of $\omega_{n}$. The expectation E[·] can be realized by averaging the result over a time window $T=L/f_{s}$. Then, by defining $E[s^{2}\left(\ell /f_{s}-\tau_{n}\right)]≜S$ and $E\left[\omega_{n}^{2}\left(\ell \right)\right]=\frac{1}{L}\sum_{\ell =0}^{L}\omega_{n}^{2}\left(\ell \right)$, we have

(3) $$h_{n}≜E\left[r_{n}^{2}\left(\ell \right)\right]=\frac{g_{n}S}{∥x_{n}{-y∥}^{\alpha }}+\frac{1}{L}\sum_{\ell =0}^{L}\omega_{n}^{2}\left(\ell \right).$$

Since $\omega_{n}∼N\left(0,\zeta_{n}^{2}\right)$, the term $\sum_{\ell =0}^{L}\omega_{n}^{2}/L$ have a $\chi^{2}$ distribution with mean $\zeta_{n}^{2}$ and variance $2\zeta_{n}^{4}/L$. According to the central limit theorem, we have $\sum_{\ell =0}^{L}\omega_{n}^{2}/L∼\mathrm{AN}\left(\zeta_{n}^{2},2\zeta_{n}^{4}/L\right)$. In order to simplify the following derivation of the source localization, we subtract a constant $\zeta_{n}^{2}$ in both side of Formula (3), i.e.,

(4) $$h_{n}-\zeta_{n}^{2}=\frac{g_{n}S}{∥x_{n}{-y∥}^{\alpha }}+\underset{\varepsilon_{n}}{\underset{︸}{\frac{1}{L}\sum_{\ell =0}^{L-1}\omega_{n}^{2}\left(\ell \right)-\zeta_{n}^{2}}}.$$

By defining $z_{n}≜h_{n}-\zeta_{n}^{2}$, we have the following average energy

(5) $$z_{n}=\frac{g_{n}S}{∥x_{n}{-y∥}^{\alpha }}+\varepsilon_{n},$$

where $\varepsilon_{n}$ is an independent random variable, denoted by $\varepsilon_{n}=\frac{1}{L}\sum_{n=0}^{L}\omega_{n}^{2}-\zeta_{n}^{2}∼\mathrm{AN}\left(0,\sigma_{n}^{2}\right)$, where $\varepsilon_{n}∼\mathrm{AN}\left(0,2\zeta_{n}^{4}/L\right)$ and $\sigma_{n}^{2}=2\zeta_{n}^{4}/L$ is the variance of $\varepsilon_{n}$. As such, the energy-based acoustic source localization can be derived according to the noisy energy readings $z_{n},\;n=1,\cdots ,N$.

From the model (5), we obtain MLE of source location y:

(6) $${\hat{y}}_{\mathrm{ML}}=\arg\;\min_{y,S}\sum_{n=1}^{N}\frac{1}{\sigma_{n}^{2}}{\left(z_{n}-\frac{g_{n}S}{∥x_{n}{-y∥}^{\alpha }}\right)}^{2}.$$

Note that the objective function is a highly nonlinear and nonconvex function of unknown y. The MLE optimization problem becomes computationally intractable. In order to avoid such an intractable computation, the Taylor series expansion of $f\left(x\right)=x^{-1/\alpha }$ on point $\frac{z_{n}}{g_{n}S}$ can be used to handle the nonconvex function. Equation (5) can be rewritten as

(7) $$\begin{array}{c} ∥x_{n}-y∥={\left(\frac{g_{n}S}{z_{n}}\right)}^{1/\alpha }+{\left(\frac{g_{n}S}{z_{n}}\right)}^{1/\alpha }\frac{\varepsilon_{n}}{\alpha z_{n}}+o\left({\left(\varepsilon_{n}\right)}^{2}\right). \end{array}$$

As such, by discarding term $o\left({\left(\varepsilon_{n}\right)}^{2}\right)$, the approximate MLE is expressed as

(8) $${\hat{y}}_{\mathrm{AML}}=\arg\min_{y,S}\sum_{n=1}^{N}\frac{z_{n}^{\left(2+2/\alpha \right)}}{g_{n}^{2/\alpha }\sigma_{n}^{2}}{\left(∥x_{n}-y∥-{\left(\frac{g_{n}S}{z_{n}}\right)}^{1/\alpha }\right)}^{2},$$

where the term $\frac{\alpha^{2}}{S^{2/\alpha }}$ is discarded since the common term is multiplied by all terms inside the summation. In the following sections, we will consider two cases including the sensor self-localization with known transmission power (or radiation power) *S* and the source localization without the knowledge of *S*.

Note that we assume the energy decay factor α is known beforehand in both the ML Formula (6) and the approximate ML Formula (8). Actually, the energy decay factor significantly depends on the surrounding acoustic environment, which can be dynamically estimated in the real scenario. An efficient method is so-called iterative optimization, which is already leveraged in [28]. The source location $\hat{y}$ and transmission power $\hat{S}$ are estimated under a given random guess of α in the first step. Then, the decay factor estimate $\hat{\alpha }$ is derived based on the logarithmic energy attenuation model under derived source location estimate y and transmission power *S*. The objective function in Equation (6) is calculated according to derived $\hat{y}$, $\hat{S}$ and $\hat{\alpha }$. If the difference between two continuous iterations is less than some threshold, we stop the iteration, or continue the iteration. In this paper, we focus on the optimization technique in the application of the sensor self-localization and the source localization. Hence, we assume that the decay factor α is known beforehand. Moreover, the estimation error of α is considered in the simulation to verify the robustness of our proposed methods.

In the application of sensor self-localization with the knowledge of the source transmission power and the source localization without the knowledge of the source transmission power, the main goal is to convert the original optimization problems into convex ones. Then, we can solve them efficiently, just as we can solve least-squares problems efficiently. With only a bit of exaggeration, we can say that, if a practical problem can be converted to a convex optimization problem, then the original problem is solved [36].

### 2.2. Real Scenario Considerations

For such a sensor self-localization and source localization system, the implementation in a real scenario by considering the communication between local nodes and the fusion center and the initialization time of the whole sensor network is analyzed here. In addition, the computational complexity of our proposed fusing algorithms will be compared in the simulation section.

*Communication complexity*: Note that the energy information of local nodes needs to be quantized and transmitted to the fusion center via wireless channel or cable medium by multiple hops for a sensor network based source localization system. The communication complexity depends on the quantization level of energy readings in local nodes. The authors in [37,38,39,40] integrated the data quantization and channel communication errors into the source localization in sensor networks, and  developed channel-aware source localization algorithms. There is no doubt that performance degrades when there are some quantization and data communication errors. In this paper, we mainly focus on how to efficiently construct the convex optimization problem based on the energy of received signal. The quantization and communication errors are not considered, which will be taken into account in the future study.

*Time complexity*: The time complexity of our proposed algorithms is related with the time of data collection and the initialization time. The data collection time depends on the sampling frequency and size. For example, the data collection time is $t=\frac{1}{48}$ s for a node with 48 kHz sampling frequency and 1000 samples. Generally, there exist time and space initialization for a sensor network, which are the first steps after the source localization system is deployed in a real scenario. The time initialization is to coarsely synchronize (the fine time synchronization for energy-based source localization system is not necessary) all the nodes and the fusion center for the energy-based source localization system. The synchronization can be efficiently fulfilled via Global Navigation Satellite System for a sensor network that can receive one pulse per second signal from satellites. The space initialization is to estimate the position of all the nodes according to the position information from the so-called anchor nodes. The time complexity depends on the network size, communication mode between sensors and the fusion center. For our proposed source localization algorithms, we can see that the localization system with network size 20 already exhibits acceptable performance from the simulation section.

## 3. Sensor Self-Localization with Known Transmission Power

For the application of sensor self-localization in a sensor network, the transmission power *S* can be known via data exchange among all the nodes. Hence, *S* can be taken as a known parameter. In this section, we first derive two kinds of sensor self-localization methods under ML criterion based on direct norm relaxation and SDR, respectively. Then, by taking colored noise into account, two source localization methods are derived under minimax criterion.

### 3.1. Sensor Self-Localization under Maximum Likelihood Criterion

The direct solving of Equation (8) is intractable due to the nonconvex objective function. Next, we transform the nonconvex MLE problem into a convex optimization one by two different relaxation techniques.

#### 3.1.1. Direct Norm Relaxation

By defining *N* auxiliary variables

(9) $$\begin{array}{c} r={\left[r_{1},\cdots ,r_{N}\right]}^{T},\;\;\;r_{n}=\frac{z_{n}^{\left(1+1/\alpha \right)}}{g_{n}^{1/\alpha }\sigma_{n}}\left(∥x_{n}-y∥-{\left(\frac{g_{n}S}{z_{n}}\right)}^{1/\alpha }\right). \end{array}$$

Equation (8) can be written as $\hat{y}=\arg\min_{y,r}\;\;{∥r∥}^{2}$, where ∥r∥ denotes the 2-norm of vector r. Note that the constraint in Equation (9) is a noncovex function of unknown y due to the term $∥x_{i}-y∥$. By introducing *N* auxiliary variables $d_{n}=∥x_{n}-y∥$, n=1,⋯,N and directly relaxing them as $∥x_{n}-y∥\leq d_{n}$, the optimization problem based on ML criterion in Equation (8) can be transformed into the following Second-order cone programming (SOCP) problem

(10) $$\begin{array}{cc} (\mathrm{ML}-\mathrm{SL}-\mathrm{DR})\;\;\;\; & \;\;\min_{y,r,\eta }\;\;\;\;\;\;\;\;\;\;\eta +\lambda_{\mathrm{MLD}}\sum_{n=1}^{N}r_{n}\\ & \mathrm{subject}\;\mathrm{to}\;\;\;\;∥\left[\begin{array}{c} 2r\\ \eta -1 \end{array}\right]∥\;\leq \;\eta +1,\;\;\;\;r={\left[r_{1},\cdots ,r_{N}\right]}^{T},\\ & \;\;\;\;\;\;\;\;\;\;\;\;\;\;\;\;\;\;\;\;\;\;\;\;\;r_{n}=\frac{z_{n}}{\sigma_{n}}\left({\left(\frac{z_{n}}{g_{n}S}\right)}^{1/\alpha }d_{n}-1\right),\\ & \;\;\;\;\;\;\;\;\;\;\;\;\;\;\;\;\;\;\;\;\;\;∥x_{n}-y∥\leq d_{n},\;\;\;n=1,\cdots ,N, \end{array}$$

where η is an epigraph variable and $\lambda_{\mathrm{MLD}}$ is a positive constant for penalization. The SOCP problem (10) is a convex problem, which is labeled as (ML-SL-DR). The notation “ML” denotes criterion *Maximum Likelihood* that is utilized, “SL” denotes *Self-Localization* and “DR” denotes the optimization problem is derived by *Direct Relaxation*. Its global optimal solution $y^{*}$ can be efficiently obtained by existing numerical tools, such as SeDuMi [41] and SDPT3 [42].

**Figure 3:**
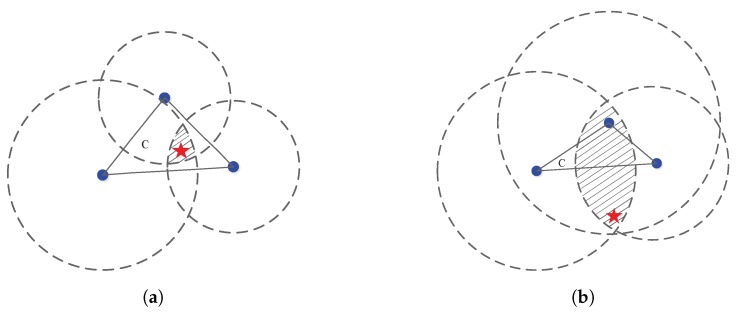
Illustration of the convex hull effect of one of the proposed alternative SOCP localization methods. The pentagram denotes the source and the solid circle denotes the sensor. (**a**) the source is located inside the convex hull (region *C*) formed by N=3 sensors; (**b**) the source is located inside the convex hull (region *C*) formed by N=3 sensors.

Note that the SOCP optimization-based sensor self-localization method directly relaxes the constraint $∥x_{n}-y∥=d_{n}$ into inequality $∥x_{n}-y∥\;\leq \;d_{n}$ in (10). For the equality constraint, the feasible region of the approximate ML problem is the *intersection point* of the circle (two-dimensional) formed by $∥x_{n}-y∥=d_{n},\;n=1,\cdots ,N$. However, for the relaxed inequality constraint, the feasible region is the *intersection region* of the disk (two-dimensional) formed by $∥x_{n}-y∥\;\leq \;d_{n},\;n=1,\cdots ,N$. Such a relaxation leads to performance degradation for the case when the source is located outside the convex hull formed by sensors. We refer to this performance degradation as *convex hull effect*. Next, by taking a planar (m=2) sensor network with N=3 nodes as an example, we state the cause of performance degradation induced by the direct norm relaxation (see Figure 3).

From the figure, we can clearly see that inequality $∥x_{n}-y∥\;\leq \;d_{n}$ denotes the disk area. The feasible region of the approximate ML problem is the intersection of *N* disk. When the source is located inside the convex hull (see region *C* in Figure 3a), the feasible region is given as the shadow area in Figure 3a. In comparison, when the source is located outside the convex hull of sensors, the feasible region is illustrated as the shadow area in Figure 3b. Comparing these two feasible regions, we can clearly see that the inside scenario (Figure 3a) has much smaller area than that of the outside scenario. As we know, the smaller the feasible region is, the more accurate the estimate is. Therefore, the relaxation from $∥x_{n}-y∥=d_{n}$ to $∥x_{n}-y∥\;\leq \;d_{n}$ leads to performance degradation when the source is moved from the inside convex hull to the outside convex hull.

#### 3.1.2. Semidefinite Relaxation

To overcome such a convex hull effect, we give another alternative SDP-based formulation for the problem of the sensor self-localization. When the source transmission power is known, the MLE in (8) can be written as the following matrix form:

(11) $$\hat{y}=\arg\min_{y}\left\{{\left(d-p\right)}^{T}Q^{-1}\left(d-p\right)\right\},$$

where d and p are defined as

(12) $$\begin{array}{cc} d & ≜{\left[∥x_{1}-y∥,\cdots ,∥x_{N}-y∥\right]}^{T},\\ p & ≜{\left[{\left(\frac{g_{1}S}{z_{1}}\right)}^{1/\alpha },\cdots ,{\left(\frac{g_{N}S}{z_{N}}\right)}^{1/\alpha }\right]}^{T}. \end{array}$$

In addition, $Q^{-1}$ is given as

(13) $$Q^{-1}=\mathrm{diag}\left(\left[\frac{z_{1}^{\left(2+2/\alpha \right)}}{g_{1}^{2/\alpha }\sigma_{1}^{2}},\frac{z_{2}^{\left(2+2/\alpha \right)}}{g_{2}^{2/\alpha }\sigma_{2}^{2}},\cdots ,\frac{z_{N}^{\left(2+2/\alpha \right)}}{g_{N}^{2/\alpha }\sigma_{N}^{2}}\right]\right),$$

where diag(·) denotes the diagonal matrix with the given vector on the main diagonal.

By defining an auxiliary matrix $D≜dd^{T}$, the objective function of (11) can be written as

(14) $$\mathrm{Tr}\left\{Q^{-1}\left(D-2dp^{T}+pp^{T}\right)\right\},$$

where Tr(·) denote the trace of a square matrix. Equation (14) holds due to the matrix property $x^{T}Ax=\mathrm{Tr}\left(Axx^{T}\right)$. It is obvious that D is semidefinite, i.e., D⪰0. According to the relation between D and d, the element $D_{nn},\;1\leq n\leq N$ can be represented as

(15) $$D_{nn}={∥x_{n}-y∥}^{2}=x_{n}^{T}x_{n}-2x_{n}^{T}y+y_{s},$$

where $y_{s}$ is defined as $y_{s}≜y^{T}y$. Moreover, by Cauchy–Schwartz inequality, the other elements can be represented as $D_{mn}=∥x_{m}-y∥\cdot ∥x_{n}-y∥\geq \left|x_{m}^{T}x_{n}-{\left(x_{m}+x_{n}\right)}^{T}y+y_{s}\right|$. Note that these constraints ($D=dd^{T}$ and $y_{s}=y^{T}y$) are still nonconvex, the solving remains difficult. The SDR technique is employed such that these two equalities can be relaxed into convex inequalities, $D⪰dd^{T}$ and $y_{s}\geq y^{T}y$, where [·]⪰0 means the matrix is semidefinite. Furthermore, these two inequalities can be rewritten as linear matrix inequalities:

(16) $$\left[\begin{array}{cc} D_{\left(N\times N\right)} & d\\ d^{T} & 1 \end{array}\right]⪰0,\;\;\left[\begin{array}{cc} I_{\left(k\times k\right)} & y\\ y^{T} & y_{s} \end{array}\right]⪰0,$$

where $I_{k\times k}$ is the k×k identity matrix, k=2 or k=3 is the dimension of position vector.

Combining all the constraints and the relaxed constraints, the original approximate ML optimization problem (8) can be written as the following SDP optimization problem:

(17) $$\begin{array}{cc} (\mathrm{ML}-\mathrm{SL}-\mathrm{SDR})\;\;\;\; & \min_{y,d,D,y_{s}}\;\;\;\;\mathrm{Tr}\left\{Q^{-1}\left(D-2dp^{T}+pp^{T}\right)\right\}+\lambda_{\mathrm{MLS}}\sum_{m=1}^{N}\sum_{n=1}^{N}D_{mn}\\ & \mathrm{subject}\;\mathrm{to}\;\;\;\;D_{nn}=x_{n}^{T}x_{n}-2x_{n}^{T}y+y_{s},\;\;\;\;D_{mn}\geq \left|x_{m}^{T}x_{n}-{\left(x_{m}+x_{n}\right)}^{T}y+y_{s}\right|,\\ & \;\;\;\;\;\;\;\;\;\;\;\;\;\;\;\;\;\;\;\;\;\;\;\;\;\;\left[\begin{array}{cc} D_{\left(N\times N\right)} & d\\ d^{T} & 1 \end{array}\right]⪰0,\;\;\;\;\left[\begin{array}{cc} I_{\left(k\times k\right)} & y\\ y^{T} & y_{s} \end{array}\right]⪰0,\\ & \;\;\;\;\;\;\;\;\;\;\;\;\;\;\;\;\;\;\;\;\;\;\;\;\;\;\;\;\;\;\;\;\;\;m,n=1,\cdots ,N,\;\;\;\;n>m, \end{array}$$

where $\lambda_{\mathrm{MLS}}>0$ is a penalization constant. The SDP problem (17) is a convex one, which can also be solved by the convex tool. Here, we label such a source localization method as (ML-SL-SDR). The denotation “SDR” denotes that the convex optimization problem is derived via a *Semidefinite Relaxation*. The same notations will be employed in the following sections, which will not be described.

Comparing the SOCP source localization method that is derived via direct norm relaxation, we observe that the key difference is the relaxed term. The nonvonvex equality $∥x_{n}-y∥=d_{n}$ is directly relaxed for (ML-SL-DR), whereas the nonconvex equality $y^{T}y=y_{s}$ and $dd^{T}=D$ are relaxed for (ML-SL-SDR).

### 3.2. Sensor Self-Localization under Minimax Criterion

The approximate ML formulation in (8) is based on the assumption of Gaussian acoustic energy reading noise. In practice, however, this assumption suffers from mismatch due to multipath environmental reverberation and echoes. The energy reading may exhibit colored noise or the statistical characteristic is unknown. By taking such uncertainty into account, the minimax criterion are considered in this section.

#### 3.2.1. Direct Norm Relaxation

By discarding the term $o\left({\left(\varepsilon_{n}\right)}^{2}\right)$, Equation (7) can be expressed as

(18) $$\alpha z_{n}{\left(\frac{z_{n}}{g_{n}S}\right)}^{1/\alpha }∥x_{n}-y∥-\alpha z_{n}=\varepsilon_{n}.$$

In the noise-free case, the right-hand side of (18) is of course zero. Thus, one approach to estimating y would be to minimize the maximum matching error. To find a globally convergent solution that is less sensitive to the noise correlation, a simplified formulation under minimax criterion can be written as

(19) $${\hat{y}}_{\mathrm{MM}}=\arg\min_{y}\max_{n=1,\cdots ,N}\left|z_{n}\left({\left(\frac{z_{n}}{g_{n}S}\right)}^{1/\alpha }∥x_{n}-y∥-1\right)\right|,$$

where “MM” denotes the optimization problem under the minimax criterion and the common term α inside absolute operation is discarded. Another perspective is to treat the minimax criterion based formulation in (19) as a Chebyshev approximation [36].

Clearly, the objective function is nonconvex of unknown y. Next, we transform the nonconvex problem into a convex one via direct norm relaxation.

Again, by introducing auxiliary variables $d_{n}=∥x_{n}-y∥,\;n=1,\cdots ,N$ and an epigraph variable γ, and directly relaxing the equality constraints into $∥x_{n}-y∥\leq d_{n}$, we have the following SOCP problem:

(20) $$\begin{array}{cc} \left(\mathrm{MM}-\mathrm{SL}-\mathrm{DR}\right) & \;\;\;\;\min_{y,d,\gamma }\;\;\;\;\;\;\;\;\;\;\gamma +\lambda_{\mathrm{MMD}}\sum_{n=1}^{N}d_{n}\\ & \mathrm{subject}\;\mathrm{to}\;\;\;\;\;\;\left|z_{n}\left({\left(\frac{z_{n}}{g_{n}S}\right)}^{1/\alpha }d_{n}-1\right)\right|\leq \gamma ,\\ & \;\;\;\;\;\;\;\;\;\;\;\;\;\;\;\;\;\;\;\;\;\;∥x_{n}-y∥\leq d_{n},\;\;n=1,\cdots ,N, \end{array}$$

where $\lambda_{\mathrm{MMD}}>0$ is the penalization factor. Such an SOCP problem in (20) is labeled as (MM-SL-DR), which can be efficiently solved to obtain the global optimal solution in polynomial time. The denotation “MM” denotes the *Minimax* criterion.

#### 3.2.2. Semidefinite Relaxation

According to the previous analysis, there exists a convex hull effect for the direct 2-norm relaxation. Correspondingly, here, we propose semidefinite relaxation based sensor self-localization method under minimax criterion. In order to avoid the direct 2-norm relaxation, square (18), we have

(21) $$\alpha^{2}z_{n}^{2}{\left(\frac{z_{n}}{g_{n}S}\right)}^{2/\alpha }{∥x_{n}-y∥}^{2}-\alpha^{2}z_{n}^{2}=\underset{\mathrm{noise}\;\nu_{n}}{\underset{︸}{\left(\varepsilon_{n}+2\alpha z_{n}\right)\varepsilon_{n}}}.$$

To find a globally convergent solution, which is less sensitive to the noise correlation, a simplified formulation under minimax criterion can be given as

(22) $${\hat{y}}_{\mathrm{MM}}=\arg\min_{y}\max_{n=1,\cdots ,N}\left|z_{n}^{2}\left({\left(\frac{z_{n}}{g_{n}S}\right)}^{2/\alpha }{∥x_{n}-y∥}^{2}-1\right)\right|,$$

where the common term $\alpha^{2}$ is discarded. As we can see that the formulation under minimax criterion is still nonconvex, it is quite amenable to be relaxed, as shown below.

We introduce auxiliary variables $d_{n}^{s}={∥x_{n}-y∥}^{2}$ and $y_{s}=y^{T}y$. Accordingly, the optimization problem (52) can be rewritten in an equivalent form

(23) $$\begin{array}{c} \min_{y,y_{s},{\left\{d_{n}^{s}\right\}}_{n=1}^{N},\gamma }\;\;\;\;\;\;\;\;\gamma \\ \mathrm{subject}\;\mathrm{to}\;\;\;\;\left|z_{n}^{2}\left({\left(\frac{z_{n}}{g_{n}S}\right)}^{2/\alpha }d_{n}^{s}-1\right)\right|\leq \gamma \\ \;\;\;\;\;\;\;\;\;\;\;\;\;\;\;\;\;\;\;d_{n}^{s}=y_{s}-2x_{n}^{T}y+x_{n}^{T}x_{n},\\ \;\;\;\;\;\;\;\;\;\;\;\;\;\;\;\;\;\;\;y_{s}=y^{T}y,\;\;n=1,\cdots ,N. \end{array}$$

However, the equation constraint $y_{s}=y^{T}y$ is not affine. To make the whole formulation convex, we relax it to inequality constraint $y_{s}\geq y^{T}y$, which can be written in a linear matrix equality. Then, we can formulate the sensor self-localization problem as

(24) $$\begin{array}{cc} (\mathrm{MM}-\mathrm{SL}-\mathrm{SDR})\;\; & \min_{y,y_{s},{\left\{d_{n}^{s}\right\}}_{n=1}^{N},\gamma }\;\;\;\;\gamma +\lambda_{\mathrm{MMS}}\sum_{n=1}^{N}d_{n}^{s}\\ & \;\;\mathrm{subject}\;\mathrm{to}\;\;\left|z_{n}^{2}\left({\left(\frac{z_{n}}{g_{n}S}\right)}^{2/\alpha }d_{n}^{s}-1\right)\right|\leq \gamma ,\\ & \;\;\;\;\;\;\;\;\;\;\;\;\;\;\;\;\;\;\;\;\;\;\;\;\;d_{n}^{s}=y_{s}-2x_{n}^{T}y+x_{n}^{T}x_{n},\\ & \;\;\;\;\;\;\;\;\;\;\;\;\;\;\;\;\;\;\;\left[\begin{array}{cc} I_{\left(k\times k\right)} & y\\ y^{T} & y_{s} \end{array}\right]⪰0,\;\;n=1,\cdots ,N, \end{array}$$

where $\lambda_{\mathrm{MMS}}>0$ is a penalization factor. We label such an SDP problem as (MM-SL-SDR), which can efficiently be solved by standard convex optimization tools.

## 4. Source Localization with Unknown Transmission Power

It sould be noted that it is intractable to obtain the transmission power for the source localization in the surveillance environment e.g., battlefield. We consider the scenario of source localization without knowledge of the transmission power.

### 4.1. Source Localization under Maximum Likelihood Criterion

In order to eliminate the unnecessary and unknown transmission power *S*, the gain ratio between the *m*th and *n*th node is defined by

(25) $$K_{mn}≜\frac{g_{m}}{g_{n}}=\frac{\left(z_{m}-\varepsilon_{m}\right){∥x_{m}-y∥}^{\alpha }}{\left(z_{n}-\varepsilon_{n}\right){∥x_{n}-y∥}^{\alpha }},$$

which can be expressed as

(26) $$∥x_{m}-y∥{\left(z_{m}-\varepsilon_{m}\right)}^{1/\alpha }=K_{mn}^{1/\alpha }∥x_{n}-y∥{\left(z_{n}-\varepsilon_{n}\right)}^{1/\alpha }.$$

The term ${\left(z_{n}-\varepsilon_{n}\right)}^{1/\alpha }$ can be approximated with Taylor series expansion to the first order and written as

(27) $${\left(z_{n}-\varepsilon_{n}\right)}^{1/\alpha }\;\approx \;z_{n}^{1/\alpha }-\frac{{z_{n}}^{\left(1/\alpha -1\right)}}{\alpha }\varepsilon_{n}.$$

Then, Equation (26) can be rewritten as

(28) $$\begin{array}{cc} ∥x_{m}-y∥z_{m}^{1/\alpha }- & ∥x_{n}-y∥z_{n}^{1/\alpha }K_{mn}^{1/\alpha }=\;\underset{\xi_{mn}}{\underset{︸}{\frac{1}{\alpha }\left(z_{m}^{\left(1/\alpha -1\right)}d_{m}\varepsilon_{m}-K_{mn}^{1/\alpha }z_{n}^{\left(1/\alpha -1\right)}d_{n}\varepsilon_{n}\right)}}, \end{array}$$

where $\xi_{mn}$ is defined as

(29) $$\xi_{mn}≜\frac{1}{\alpha }\left(z_{m}^{\left(1/\alpha -1\right)}d_{m}\varepsilon_{m}-K_{mn}^{1/\alpha }z_{n}^{\left(1/\alpha -1\right)}d_{n}\varepsilon_{n}\right).$$

To derive the ML estimate of unknown source location y, we have to analytically derive the covariance matrix Σ of noises $\xi_{mn},\;1\leq m\leq N,\;1\leq n\leq N,\;m\neq n$. The common sensors in the gain ratio of all pairwise measurements leads to correlated noise. By defining the noise vector as

(30) $$\xi ={\left[\xi_{12},\cdots ,\xi_{1N},\xi_{23},\cdots ,\xi_{2N},\xi_{31},\cdots ,\xi_{N-1,N}\right]}^{T},$$

where the dimension is M=N(N−1)/2. In (29), by replacing $d_{n}$ with (7), we derive the element of the mean of ξ is

(31) $$E\left[\xi_{\ell }\right]=E\left[\xi_{mn}\right]=\frac{{\left(g_{m}S\right)}^{1/\alpha }\sigma_{m}^{2}}{\alpha^{2}z_{m}^{2}}-\frac{{\left(K_{mn}g_{n}S\right)}^{1/\alpha }\sigma_{n}^{2}}{\alpha^{2}z_{n}^{2}},$$

where 1≤ℓ≤M is the index of vector ξ. The element of covariance matrix Σ is

(32) $$\begin{array}{c} \Sigma_{\iota \kappa }=E\left[\left(\xi_{mn}-E\left[\xi_{mn}\right]\right)\left(\xi_{ij}-E\left[\xi_{ij}\right]\right)\right]=E\left[\xi_{mn}\xi_{ij}\right]-E\left[\xi_{mn}\right]E\left[\xi_{ij}\right]. \end{array}$$

The second order moment $E[\xi_{mn}\xi_{ij}]$ can further be written as

(33) $$\begin{array}{cc} E[\xi_{mn}\xi_{ij}]= & \;\frac{1}{\alpha^{2}}\left[{\left(z_{m}z_{i}\right)}^{1/\alpha -1}E\left[d_{m}\varepsilon_{m}d_{i}\varepsilon_{i}\right]\right.\left.+{\left(K_{mn}K_{ij}\right)}^{1/\alpha }{\left(z_{n}z_{j}\right)}^{1/\alpha -1}E\left[d_{n}\varepsilon_{n}d_{j}\varepsilon_{j}\right]\right.\\ & \left.-K_{mn}^{1/\alpha }{\left(z_{n}z_{i}\right)}^{1/\alpha -1}E\left[d_{n}\varepsilon_{n}d_{i}\varepsilon_{i}\right]\right.\left.-K_{ij}^{1/\alpha }{\left(z_{j}z_{m}\right)}^{1/\alpha -1}E\left[d_{m}\varepsilon_{m}d_{j}\varepsilon_{j}\right]\right]. \end{array}$$

According to the approximation in (7), the term $E[d_{u}\varepsilon_{u}d_{v}\varepsilon_{v}]$ for u=m,n and v=i,j can be derived according to the following different conditions:

- when u=v, the term $E[d_{u}\varepsilon_{u}d_{v}\varepsilon_{v}]$ is
  (34) $$E\left[d_{u}\varepsilon_{u}d_{v}\varepsilon_{v}\right]={\left(\frac{g_{u}S}{z_{u}}\right)}^{2/\alpha }\left(\sigma_{u}^{2}+\frac{2\sigma_{u}^{4}}{\alpha^{2}z_{u}^{2}}\right),$$
- when u≠v, the term $E[d_{u}\varepsilon_{u}d_{v}\varepsilon_{v}]$ is
  (35) $$E\left[d_{u}\varepsilon_{u}d_{v}\varepsilon_{v}\right]={\left(\frac{g_{u}S}{z_{u}}\right)}^{1/\alpha }\frac{\sigma_{u}^{2}}{\alpha z_{u}}{\left(\frac{g_{v}S}{z_{v}}\right)}^{1/\alpha }\frac{\sigma_{v}^{2}}{\alpha z_{v}},$$

where $\sigma_{u}^{2}$ is the variance of acoustic energy reading noise $\varepsilon_{u}$ in (5).

Hence, M=N(N−1)/2 equations of (28) can be written as a vector form

(36) Gd=ξ,

where G is

(37) $$G=\left[\begin{array}{ccccccc} z_{1}^{\frac{1}{\alpha }} & -{\left(K_{12}z_{2}\right)}^{\frac{1}{\alpha }} & 0 & 0 & \cdots & 0 & 0\\ z_{1}^{\frac{1}{\alpha }} & 0 & -{\left(K_{13}z_{3}\right)}^{\frac{1}{\alpha }} & 0 & \cdots & 0 & 0\\ \vdots & \vdots & \vdots & \vdots & & \vdots & \\ z_{1}^{\frac{1}{\alpha }} & 0 & 0 & 0 & \cdots & 0 & -{\left(K_{1N}z_{N}\right)}^{\frac{1}{\alpha }}\\ 0 & z_{2}^{\frac{1}{\alpha }} & -{\left(K_{23}z_{3}\right)}^{\frac{1}{\alpha }} & 0 & \cdots & 0 & 0\\ 0 & z_{2}^{\frac{1}{\alpha }} & 0 & -{\left(K_{24}z_{4}\right)}^{\frac{1}{\alpha }} & \cdots & 0 & 0\\ \vdots & \vdots & \vdots & \vdots & & \vdots & \vdots \\ 0 & z_{2}^{\frac{1}{\alpha }} & 0 & 0 & \cdots & 0 & -{\left(K_{2N}z_{N}\right)}^{\frac{1}{\alpha }}\\ \vdots & \vdots & \vdots & \vdots & & \vdots & \vdots \\ 0 & 0 & 0 & 0 & \cdots & z_{N-1}^{\frac{1}{\alpha }} & -{\left(K_{N-1,N}z_{N}\right)}^{\frac{1}{\alpha }} \end{array}\right],$$

and vector d is

(38) $$d={\left[∥x_{1}-y∥,∥x_{2}-y∥,\cdots ,∥x_{N}-y∥\right]}^{T}.$$

ξ is given in (30). The dimension of G is M×N. According to (36) with unknown source location y, the ML estimation can be expressed as

(39) $${\hat{y}}_{\mathrm{ML}}=\min_{y,d}\;\;{\left(Gd\right)}^{T}\Sigma^{-1}\left(Gd\right),$$

where Σ is the covariance matrix of noise vector ξ, which can be derived according to (31)∼(35). Please note that there exists an unknown term $S^{2/\alpha }$ in the covariance matrix Σ in (32), which can be discarded in the optimization (36), since it is not necessarily exactly known and is independent of the unknown vector d.

As such, the source localization with unknown transmission power *S* is formulated as (39) according to the gain ratio or energy ratio under ML criterion.

Note that the ML source localization problem in (36) is a nonlinear optimization problem. It is difficult to solve such a nonconvex optimization problem. The objective function of (39) can be rewritten as

(40) $$\begin{array}{cc} \varphi & ≜\mathrm{Tr}\left(\Sigma^{-1}GDG^{T}\right), \end{array}$$

where D is defined as $D≜dd^{T}$. Based on such a new matrix variable, we will pursue a convex relaxation via direct norm relaxation and SDR, respectively, to generate convex optimization problems, which can be efficiently solved in polynomial time.

#### 4.1.1. Direct Norm Relaxation

First, we consider the direct norm relaxation. The equality constraint of $D≜dd^{T}$ is not an affine set. Such an equality can be relaxed into $D⪰dd^{T}$, which can be written as

(41) $$\begin{array}{c} \left[\begin{array}{cc} D_{N\times N} & d\\ d^{T} & 1 \end{array}\right]⪰0. \end{array}$$

After such a relaxation, there are still *N* nonconvex constraints in (38). Again, the nonconvex equation can be directly relaxed as $d={\left[d_{1},\cdots ,d_{N}\right]}^{T},\;\;∥x_{n}-y∥\leq d_{n}$. The optimization problem based on ML criterion in (39) can be formulated as

(42) $$\begin{array}{cc} (\mathrm{ML}-\mathrm{RL}-\mathrm{DR})\;\; & \;\;\min_{y,D,d}\;\;\;\;\;\mathrm{Tr}\left(\Sigma^{-1}GDG^{T}\right)+\beta_{\mathrm{MLD}}\sum_{m=1}^{N}\sum_{n=1}^{N}D_{mn}\\ & \;\;\;\;\mathrm{subject}\;\mathrm{to}\;\;\;\;\left[\begin{array}{cc} D_{N\times N} & d\\ d^{T} & 1 \end{array}\right]⪰0,\;\;\;\;d={\left[d_{1},\cdots ,d_{N}\right]}^{T},\\ & \;\;\;\;\;\;\;\;\;\;\;\;\;\;\;\;\;\;\;\;\;\;∥x_{n}-y∥\leq d_{n},\;\;\;\;n=1,\cdots ,N, \end{array}$$

where $\beta_{\mathrm{MLD}}>0$ is a penalization factor. We label such an optimization problem as (ML-RL-DR). The denotation “RL” denotes *souRce Localization*.

#### 4.1.2. Semidefinite Relaxation

Similar to the scenario of the sensor self-localization with known transmission power, by defining $y_{s}=y^{T}y$, we have following convex constraints $D_{nn}={∥x_{n}-y∥}^{2}=x_{n}^{T}x_{n}-2x_{n}^{T}y+y_{s}$ and $D_{mn}=∥x_{m}-y∥\cdot ∥x_{n}-y∥\geq \left|x_{m}^{T}x_{n}-{\left(x_{m}+x_{n}\right)}^{T}y+y_{s}\right|$. Similarly, the equality constraints $y_{s}=y^{T}y$ can be relaxed as $y_{s}\geq y^{T}y$, which is equivalently written as a linear matrix equality. Combining all the constraints and the relaxed constraints, the original ML problem (39) can be written as the following SDP optimization problem:

(43) $$\begin{array}{cc} \left(\mathrm{ML}-\mathrm{RL}-\mathrm{SDR}\right) & \;\;\;\min_{y,D,y_{s}}\;\;\mathrm{Tr}\left(GDG^{T}\Sigma^{-1}\right)+\beta_{\mathrm{MLS}}\sum_{m=1}^{N}\sum_{n=1}^{N}D_{mn}\\ & \mathrm{subject}\;\mathrm{to}\;\;\;\;\;\;\;\;D_{nn}=x_{n}^{T}x_{n}-2x_{n}^{T}y+y_{s},\\ & \;\;\;\;\;\;\;\;\;\;\;\;\;\;\;\;\;D_{mn}\geq \left|x_{m}^{T}x_{n}-{\left(x_{m}+x_{n}\right)}^{T}y+y_{s}\right|,\\ & \;\;\;\;\;\;\;\;\;\;\;\;\;\;\;\;\left[\begin{array}{cc} I_{\left(k\times k\right)} & y\\ y^{T} & y_{s} \end{array}\right]⪰0,\;\;D_{\left(N\times N\right)}⪰0,\\ & \;\;\;\;\;\;\;\;\;\;\;\;\;\;\;\;\;\;m=1,\cdots ,N,\;\;n=1,\cdots ,N, \end{array}$$

where $\beta_{\mathrm{MLS}}>0$ is a penalization factor. Such an SDP optimization problem labeled as (ML-RL-SDR), which can be efficiently solved by existing numerical tools.

### 4.2. Source Localization under Minimax Criterion

The (ML-RL-SDR) optimization problem in (43) is an SDP-based convex optimization problem for ML estimate in (39). We can see that the ML estimate relies on the known covariance matrix Σ. Similarly, the minimax criterion is considered by taking colored acoustic energy noise into account.

#### 4.2.1. Direct Norm Relaxation

According to (28), the source localization under minimax criterion can be written as

(44) $${\hat{y}}_{\mathrm{MM}}=\arg\min_{y}\max_{\begin{array}{c} m,n=1,\cdots ,N\\ n>m \end{array}}\left|∥x_{m}-y∥z_{m}^{1/\alpha }-∥x_{n}-y∥z_{n}^{1/\alpha }K_{mn}^{1/\alpha }\right|.$$

Such an optimization can be expressed as

(45) $$\begin{array}{c} \;\;\;\min_{y,d}\;\;\;\;\;{∥Gd∥}_{\infty }\\ \mathrm{subject}\;\mathrm{to}\;\;\;\;∥x_{n}-y∥=d_{n},\;\;n=1,\cdots ,N, \end{array}$$

where G and d are defined in (37) and (38), and ${∥\cdot ∥}_{\infty }$ is the $\ell_{\infty }$ norm. We can see that the equality constrain in (45) is nonconvex. The equality constrain can be directly relaxed as a convex one $∥x_{n}-y∥\leq d_{n}$. Thus, we obtain following optimization problem:

(46) $$\begin{array}{cc} (\mathrm{MM}-\mathrm{RL}-\mathrm{DR})\;\; & \;\;\;\min_{y,d}\;\;{∥Gd∥}_{\infty }+\beta_{\mathrm{MMD}}\sum_{n=1}^{N}d_{n}\\ & \mathrm{subject}\;\mathrm{to}\;\;\;∥x_{n}-y∥\leq d_{n},\;\;n=1,\cdots ,N, \end{array}$$

where $\beta_{\mathrm{MMD}}>0$ is a constant for penalization. Such an optimization problem is convex in the variables d and y. We label such a convex optimization as (MM-RL-DR), which can be solved by convex optimization tools.

#### 4.2.2. Semidefinite Relaxation

Such direct relaxation source localization suffers from convex hull effect, which exhibits dramatically performance degradation when the source is located outside the convex hull formed by sensors. Hence, an SDP-based source localization is proposed under minimax criterion. The formulation (28) can be rewritten as $∥x_{m}-y∥z_{m}^{1/\alpha }=∥x_{n}-y∥z_{n}^{1/\alpha }K_{mn}^{1/\alpha }+\xi_{mn}$, which leads to

(47) $$\begin{array}{c} ∥x_{m}{-y∥}^{2}z_{m}^{2/\alpha }-{∥x_{n}-y∥}^{2}z_{n}^{2/\alpha }K_{mn}^{2/\alpha }\\ \;=\underset{\varsigma_{mn}}{\underset{︸}{\xi_{mn}\left(\xi_{mn}+2∥x_{n}-y∥z_{n}^{1/\alpha }K_{mn}^{1/\alpha }\right)}}. \end{array}$$

Similarly, in the noise-free case, the right-hand side of (47) is zero, we obtain a simplified formulation by adopting the minimax criterion

(48) $${\hat{y}}_{\mathrm{MM}}=\arg\min_{y}\max_{\begin{array}{c} m,n=1,\cdots ,N\\ n>m \end{array}}\left|∥x_{m}{-y∥}^{2}z_{m}^{2/\alpha }-{∥x_{n}-y∥}^{2}z_{n}^{2/\alpha }K_{mn}^{2/\alpha }\right|.$$

Such a formulation is still nonconvex. Next, the SDR technique is adopted to relax the nonconcex constraints to convex ones.

By defining auxiliary variables $d_{n}^{s}≜{∥x_{n}-y∥}^{2}$ and $d^{s}≜{\left[d_{1}^{s},d_{2}^{s},\cdots ,d_{N}^{s}\right]}^{T}$, the objective function can be equivalently written as

(49) $$\min_{d^{s},y}\;\;{∥G^{s}d^{s}∥}_{\infty },$$

where $G^{s}$ is defined as

(50) $$G^{s}=\left[\begin{array}{ccccccc} z_{1}^{\frac{2}{\alpha }} & -{\left(K_{12}z_{2}\right)}^{\frac{2}{\alpha }} & 0 & 0 & \cdots & 0 & 0\\ z_{1}^{\frac{2}{\alpha }} & 0 & -{\left(K_{13}z_{3}\right)}^{\frac{2}{\alpha }} & 0 & \cdots & 0 & 0\\ \vdots & \vdots & \vdots & \vdots & & \vdots & \\ z_{1}^{\frac{2}{\alpha }} & 0 & 0 & 0 & \cdots & 0 & -{\left(K_{1N}z_{N}\right)}^{\frac{2}{\alpha }}\\ 0 & z_{2}^{\frac{2}{\alpha }} & -{\left(K_{23}z_{3}\right)}^{\frac{2}{\alpha }} & 0 & \cdots & 0 & 0\\ 0 & z_{2}^{\frac{2}{\alpha }} & 0 & -{\left(K_{24}z_{4}\right)}^{\frac{2}{\alpha }} & \cdots & 0 & 0\\ \vdots & \vdots & \vdots & \vdots & & \vdots & \vdots \\ 0 & z_{2}^{\frac{2}{\alpha }} & 0 & 0 & \cdots & 0 & -{\left(K_{2N}z_{N}\right)}^{\frac{2}{\alpha }}\\ \vdots & \vdots & \vdots & \vdots & & \vdots & \vdots \\ 0 & 0 & 0 & 0 & \cdots & z_{N-1}^{\frac{2}{\alpha }} & -{\left(K_{N-1,N}z_{N}\right)}^{\frac{2}{\alpha }} \end{array}\right].$$

By expanding the nonconvex constraint $d_{n}^{s}={∥x_{n}-y∥}^{2}$ and defining $y_{s}=y^{T}y$, we have

(51) $$d_{n}^{s}=x_{n}^{T}x_{n}-2x_{n}^{T}y+y_{s},$$

which is an affine function of $d_{n}^{s}$, y and $y_{s}$. The nonconvex constraint $y^{T}y$ can be relaxed as $y_{s}\geq y^{T}y$, which can be written as a linear matrix equality. By combining all the convex constraints and semidefinite relaxation, the source localization under minimax criterion can be transformed as the following convex optimization problem:

(52) $$\begin{array}{cc} \left(\mathrm{MM}-\mathrm{RL}-\mathrm{SDR}\right)\;\;\;\min_{d^{s},y} & \;\;\;\;{∥G^{s}d^{s}∥}_{\infty }+\beta_{\mathrm{MMS}}\sum_{n=1}^{N}d_{n}^{s}\\ \mathrm{subject}\;\mathrm{to} & \;\;\;\;\;\;\;\;\;\left[\begin{array}{cc} I_{\left(k\times k\right)} & y\\ y^{T} & y_{s} \end{array}\right]⪰0,\\ & \;d_{n}^{s}=y_{s}-2x_{n}^{T}y+x_{n}^{T}x_{n},\;\;n=1,\cdots ,N, \end{array}$$

where $\beta_{\mathrm{MMS}}>0$ is a positive constant for penalization. Such a convex optimization is labeled as (MM-RL-SDR).

## 5. Cramér–Rao Low Bound (CRLB) of the Energy-Based Source Localization

In this section, CRLBs of the energy-based acoustic source localization with known and unknown transmission power (or radiation power) are given under any energy decay factor.

The Fisher information matrix is

(53) $$J=E\left\{\left[\frac{\partial \ln p(Z|\theta^{\prime })}{\partial \theta^{\prime }}\right]{\left[\frac{\partial \ln p(Z|\theta^{\prime })}{\partial \theta^{\prime }}\right]}^{T}\right\},$$

where $\theta^{\prime }$ is the unknown vector. For the sensor self-localization case with known *S*, $\theta^{\prime }=y$, for the source localization case with unknown *S*, $\theta^{\prime }={\left[y^{T},S\right]}^{T}$.

According to the original ML problem in (6), for the sensor self-localization with known *S*, we have

(54) $$J_{\mathrm{SL}}=B^{T}B,$$

where $B={\left[\frac{\alpha g_{1}S\left(x_{1}-y\right)}{\sigma_{1}d_{1}^{\alpha +2}},\frac{\alpha g_{2}S\left(x_{2}-y\right)}{\sigma_{2}d_{2}^{\alpha +2}},\cdots ,\frac{\alpha g_{N}S\left(x_{N}-y\right)}{\sigma_{N}d_{N}^{\alpha +2}}\right]}^{T}$. Note that $J_{\mathrm{SL}}$ is a *m*-dimensional matrix. Thus, the lower bound of the source localization error for known *S* can be calculated as follows:

(55) $$\begin{array}{cc} {\mathrm{CRLB}}_{\mathrm{SL}} & =\sqrt{{\left[J_{\mathrm{SL}}^{-1}\right]}_{11}+\cdots +{\left[J_{\mathrm{SL}}^{-1}\right]}_{mm}}=\sqrt{\mathrm{Tr}\left(J_{\mathrm{SL}}^{-1}\right)}, \end{array}$$

where “SL” denotes the sensor self-localization scenario with known *S*.

For unknown *S*, the Fisher information is

(56) $$J_{\mathrm{RL}}=\left[\begin{array}{c} B^{T}\\ R^{T} \end{array}\right]\left[\begin{array}{cc} B & R \end{array}\right],$$

where $R={\left[\frac{g_{1}}{\sigma_{1}d_{1}^{\alpha }},\frac{g_{2}}{\sigma_{2}d_{2}^{\alpha }},\cdots ,\frac{g_{N}}{\sigma_{N}d_{N}^{\alpha }}\right]}^{T}$. Thus, the lower bound of the the source localization error for unknown *S* is

(57) $${\mathrm{CRLB}}_{\mathrm{RL}}=\sqrt{{\left[J_{\mathrm{RL}}^{-1}\right]}_{11}+\cdots +{\left[J_{\mathrm{RL}}^{-1}\right]}_{mm}},$$

where “RL” denotes the source localization scenario with unknown *S*.

## 6. Simulation Results

In this section, several examples and the corresponding performance analysis are given to compare the proposed methods with the existing ones. All of the convex optimization problems are solved by the MATLAB CVX package (Version 2.1, CVX Research, Inc., Austin, TX, USA) [43], where the solver is SeduMi CVX. Note that some source localization methods in literature refine the results of the convex optimization problem to improve the overall performance [27]. Equivalently, the source localization procedure can be concluded as two step: (1) the solution of the convex optimization problem is derived, and (2) a refinement method, e.g., randomization procedure, is carried out based on the initial solution. In this section, to make a fair comparison of different source localization optimization methods, we carry out the performance comparison without additional refinement, i.e., the results are derived from the only optimization step.

In our simulations, if the sensor locations are fixed, we place N=12 sensors in a two-dimensional area at

(58) $$\begin{array}{c} x_{1}=\left(40,40\right),x_{2}=\left(40,-40\right),x_{3}=\left(-40,40\right),\\ x_{4}=\left(-40,-40\right),x_{5}=\left(40,0\right),x_{6}=\left(0,40\right),\\ x_{7}=\left(-40,0\right),x_{8}=\left(0,-40\right),x_{9}=\left(20,40\right),\\ x_{10}=\left(20,-40\right),x_{11}=\left(-20,40\right),x_{12}=\left(-20,-40\right). \end{array}$$

The performance is evaluated in terms of the root-mean-square error (RMSE), which is defined as $\mathrm{RMSE}=\sqrt{\sum_{\bar{m}=1}^{\bar{M}}\frac{∥{\hat{y}}_{\hat{m}}-y_{t}{∥}^{2}}{\bar{M}}}$, where ${\hat{y}}_{\hat{m}}$ is the estimate of the source position in the $\hat{m}$th Monte Carlo run, and $\bar{M}$ is the number of the Monte Carlo runs. According to (1), the source transmission or radiation signal to noise ratio (SNR) is defined as $\mathrm{SNR}=\frac{S}{\zeta_{n}^{2}}$. In this paper, we assume the identical noise variance, i.e., $\zeta_{n}=\zeta$ for n=1,⋯,N and identical gain factor, i.e., $g_{n}=g=1$ for n=1,⋯,N. The  numbers of samples *L* for each node are assumed to be identical. Here, we consider four simulation examples for both the sensor self-localization and the source localization. The first example is to select the proper penalization factors for different source localization methods. The performance comparison under different geometric layouts is carried out in the second example. The third example analyzes the robust performance against the estimated error of the energy decay factor, which is iteratively estimated via person-by-person optimization. The performance comparison against different SNRs, numbers of sensors *N*, number of samples *L*, as well as the energy decay factor α, is given in the last example.

### 6.1. Performance Analysis of the Sensor Self-Localization

In comparison, the approximate ML estimator, i.e., the solution of formulation (8) for the sensor self-localization with known *S* is compared with our proposed methods. Such an approximate estimator is solved by MATLAB function fminsearch, which uses the derivative-free method [44]. As we know, the ML estimator suffers from the convergence problem and the result can end up with a local optimum or a saddle point due to inappropriate initialization. Here, we consider two different cases with two kinds of initial values: one is taking the exact source location as the initial point (labeled as ML-searching-T), and the other is taking a point far away from the true source location as the initial point (labeled as ML-searching-F).

**Figure 4:**
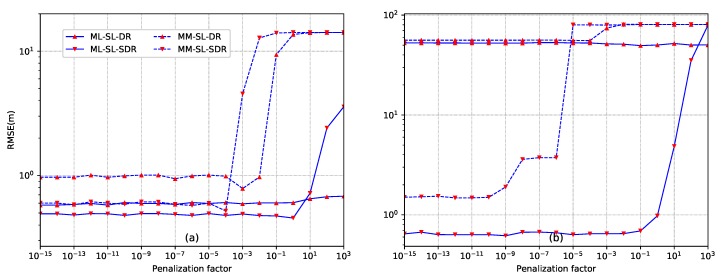
Comparison of the proposed source localization methods with different penalization factors. (**a**) the source is inside the convex hull of the sensor nodes ($y={\left[10,10\right]}^{T}$); (**b**) the source is outside the convex hull of the sensor nodes ($y={\left[10,80\right]}^{T}$).

*Example 1: selection of the penalization factor*. To illustrate the importance of the penalization factor for our proposed methods, this example examines RMSE versus the penalization factor. The results are given in Figure 4. N=12 sensors are distributed according to setup (58). For Figure 4a, the source is located at $y={\left[10,10\right]}^{T}$. The transmission power is set as S=1000 and SNR is SNR =30 dB. The sampling size is L=1000 and energy decay factor is α=1.8. For Figure 4b, the source is located at $y={\left[10,80\right]}^{T}$. The transmission power is S=1000 and SNR is SNR =40 dB. The sampling size is also L=1000 and the energy decay factor is α=1.8. We can see that the optimal penalization factor depends on the spatial relationship between nodes and the source. When the source is inside the convex hull of sensors, the penalization weight can be selected in the range of $\left[10^{-15},10^{-5}\right]$. When the source is outside the convex hull of sensors, the penalization factor can be selected in the range of $\left[10^{-15},10^{-10}\right]$. In the following examples, we will select $\alpha_{\mathrm{MLD}}=\alpha_{\mathrm{MLS}}=\alpha_{\mathrm{MMD}}=\alpha_{\mathrm{MMS}}=10^{-14}$ for the case of inside convex hull, and set all the penalization factors as $10^{-13}$ for the case of outside convex hull.

*Example 2: performance analysis with different geometric layouts*. It has been shown that the geometric layout of the sensors and the source has a significant impact on the localization performance [21]. In order to reflect such an impact, we keep the sensor locations fixed (N=12) according to the setup (58). For the first case, the source is placed at the point $y={\left[10,10\right]}^{T}$, which is inside the convex hull formed by the twelve sensors. The energy decay factor is α=1.8. In the second case, the source is placed at the point $y={\left[10,80\right]}^{T}$, which is outside the convex hull formed by the sensors. The energy decay factor is α=1.6. For both of these two cases, the source transmission power is S=1000, and the sampling size is L=1000. The noises are generated as independent and identically distributed (i.i.d) Gaussian and *S* is assumed to be known with S=1000. The number of Monte Carlo runs is $\bar{M}=1000$.

**Figure 5:**
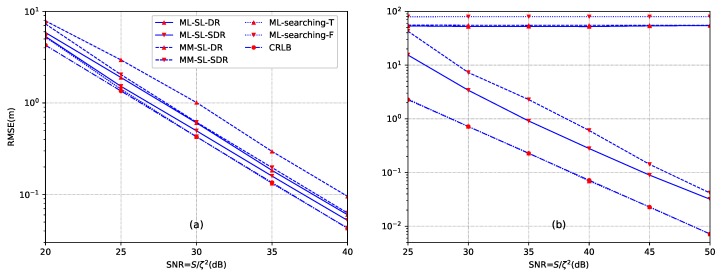
Comparison of searching method of ML and the proposed methods. (**a**) the source is located at $y={\left[10,10\right]}^{T}$; (**b**) the source is located at $y={\left[10,80\right]}^{T}$.

Figure 5 illustrates the performance of different methods versus the SNR by using the setup (58). The CRLB is also included in the figure. From Figure 5a, we can see that the proposed ML-SL-SDR method performs optimally (i.e., achieves approximately the corresponding CRLB) for a wide range of SNR. The performance of ML-SL-SDR and MM-SL-SDR is very close to each other, and both of them generate superior performance than MM-SL-DR from low SNR to high SNR when the source is located at ${\left[10,10\right]}^{T}$. Two ML searching methods generate the same results in such a case, which provide very close performance to the CRLB. In comparison, when the source is located outside the convex hull (see Figure 5b), the proposed ML-SL-DR and MM-SL-DR, as well as the ML-searching-F with an improper initial value fail to give a good estimate. As has been analyzed, this is because that the direct relaxation from $∥x_{n}-y∥=d_{n}$ to $∥x_{n}-y∥\;\leq \;d_{n}$ leads to wide feasible region compared with the same relaxation technique when the source is inside the convex hull. ML-searching-F is unable to escape a local minimum due to inappropriate initial value. In this case, the performance of ML-searching-T method is very close to the CRLB of the acoustic energy-based model.

**Figure 6:**
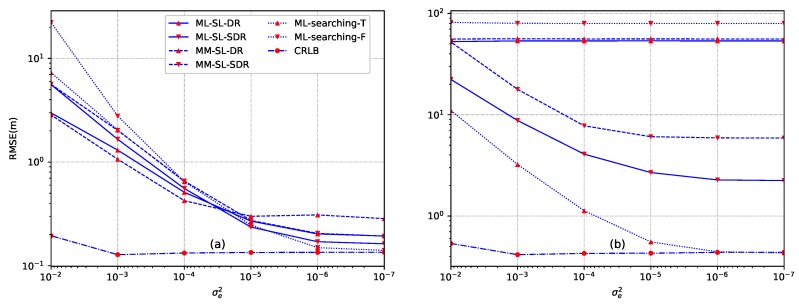
Comparison of searching method of ML and the proposed methods against estimated error of the energy decay factor. (**a**) the source is located at $y={\left[10,10\right]}^{T}$; (**b**) the source is located at $y={\left[10,80\right]}^{T}$.

*Example 3: performance analysis against the estimated error of the energy decay factor.* In previous examples, we assume that the energy decay factor α is accurately known. However, the estimation still has some errors. Thus, in this example, we examine the robust performance against the estimated error of the energy decay factor. We assume that the estimate of the energy decay factor is known as

(59) $$\hat{\alpha }=\alpha +\Delta \alpha ,$$

where α is the truth energy decay factor and Δα is the estimated error, which follows a truncated Gaussian distribution in the interval a≤α≤b. The pdf is given as

(60) $$f\left(\Delta \alpha \right)=\left\{\begin{array}{cc} \frac{\exp\left(-\frac{\Delta \alpha }{2\sigma_{e}^{2}}\right)}{\sigma_{e}\left(\Phi \left(\frac{b}{\sigma_{e}}\right)-\Phi \left(\frac{a}{\sigma_{e}}\right)\right)}, & a\leq \Delta \alpha \leq b\\ 0, & \mathrm{others}, \end{array}\right.,$$

where $\sigma_{e}^{2}$ is the variance of the truncated Gaussian distribution and Φ(·) is the cumulative distribution function of the standard norm distribution. The results against energy decay factor errors in terms of different variances for the case of both inside convex hull and outside convex hull are given in Figure 6, respectively. The truth of the energy decay factor is α=1.8 and the truncated parameters are a=1.5 and b=2.1. N=12 sensors are deployed according to setup (58). For Figure 6a, the source is located at $y={\left[10,10\right]}^{T}$, For Figure 6b, the source is located at $y={\left[10,80\right]}^{T}$. For both of the two cases, the source transmission power is set as S=1000, SNR is SNR = 35 dB, and the length of sampling is L=1000.

From Figure 6a, when the source is located inside the convex hull of sensors, the proposed direct norm relaxation based methods (ML-SL-DR and MM-SL-DR) provide better performance than the other proposed methods as well as the two ML-based searching methods at higher $\sigma_{e}^{2}$ ($\sigma_{e}^{2}\geq 10^{-4}$). However, the performance of MM-SL-DR degrades with a higher rate when $\sigma_{e}^{2}$ decreases, and finally converges to a fixed value. When the source is located outside the convex hull of sensors (see Figure 6b), the ML-searching-T by utilizing the exact location as the initial value shows superior performance against the estimated error of the energy decay factor. However, the requirement is quite challenging and impractical. The proposed ML-SL-SDR method exhibits better performance than MM-SL-SDR. Similar to the result of *Example 2*, the two kinds of direct norm relaxation based source localization methods fail to work due to the larger feasible region than that of the case of inside convex hull.

**Figure 7:**
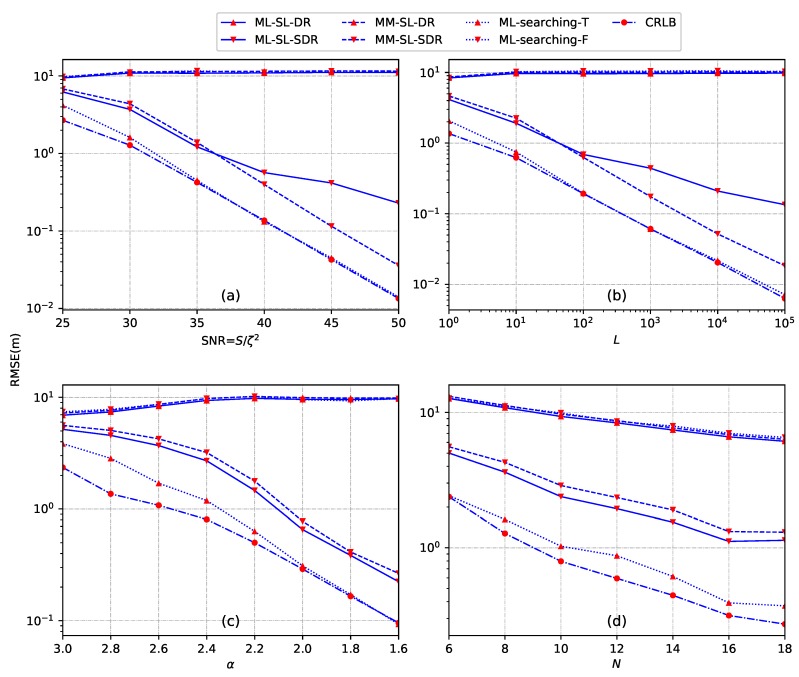
Performance analysis when the source is located randomly in a region of size 50 × 50 $m^{2}$. (**a**) RMSE versus SNR; (**b**) RMSE versus *L*; (**c**) RMSE versus α; (**d**) RMSE versus *N*.

*Example 4: sensitivity to the model parameters.* According to the energy decay model in (5), the acoustic energy reading of each sensor depends on the number of samples *L*, the energy decay factor α, the source transmission power *S* and the noise variance $\zeta^{2}$. In this example, the impact on the source localization performance of these parameters as well as the number of sensors *N* is examined. We consider a two-dimensional sensor network with *N* sensors deployed in a region of size 50 × 50 $m^{2}$. The sensors and the source are randomly and uniformly distributed in this region. In Figure 7, we plot the performance of different source localization methods versus SNR, the sample number *L*, energy decay factor α and the number of sensors *N*, respectively, by averaging over all estimated source locations and noise realizations. In this example, we set the source radiated power is S=500 and the number of Monte Carlo runs is $\bar{M}=5000$. In Figure 7a, we set L=500, α=2.4, and N=8. In Figure 7b, we set SNR =40 dB, α=2.4, and n=10. In Figure 7c, we set SNR =30 dB, L=500, and N=10. In Figure 7d, we set SNR =30 dB, L=500, and α=2.4.

It can be seen that the ML-searching-T method by taking the true source location as the initial value provides the best performance among all the methods, which is very close to the CRLB for higher SNR (SNR ≥35 dB), larger number of sampling *L* ($L\geq 10^{2}$), as well as smaller energy decay factor α (α≤2). The performance of the proposed ML-SL-SDR method is slightly better than the MM-SL-SDR method for lower SNR (SNR ≤35 dB) and smaller *L* ($L\leq 10^{2}$), and for the whole range of α and *N*. Similar to the previous examples, both of the proposed ML-SL-DR and MM-SL-DR, as well as the ML-searching-F methods, fail to give a good estimate. This is due to the direct relaxation and the inappropriate initial value. Moreover, a significant gap exists between the convex optimization based source localization methods and CRLB. This observation illustrates that there still exists room for significant improvement in the acoustic energy based model.

*Complexional computation:* To illustrate the computational cost of our proposed sensor self-localization algorithms, we give a comparison of the average CPU time. All the results are obtained by using an Intel Xeon core E5-1630 PC (Singapore) with 3.7 GHz CPU and 16 G RAM. The comparison of the computational complex of our proposed sensor self-localization algorithms as well as the ML searching algorithm is given in Table 1. It should be noted that the ML-Searching algorithms are based on the local minimum searching, which runs less CPU time than the convex optimization based algorithms. From the results, we can see that the proposed SDR-based source localization algorithms run less time than DR-based algorithms.

*Simulation summary:* When the source transmission power (or radiation power) *S* is known for the sensor self-localization, we propose two kind source localization methods via direct norm relaxation and SDR under ML and minimax criterion, respectively. From above simulations, we have the following conclusions:

- The proposed SDR-based source localization methods, especially ML-SL-SDR, provide superior performance for the cases of both inside convex hull formed by sensors and outside convex hull. ML-SL-SDR exhibits better performance than MM-SL-SDR since the distribution of energy noise is utilized to improve the accuracy of source localization.
- For the case of inside convex hull, the proposed direct norm relaxation based methods provide a robust estimate against the estimated error of the energy decay factor noise. For the case of outside convex hull, SDR-based methods can give robust source location estimate.
- When the sensors and the source are randomly and uniformly distributed in a square region, the proposed MM-SL-SDR provides robust performance in a wide range of SNR, sampling number *L*, energy decay factor α and number of sensors *N*. Moreover, ML-SL-SDR outperforms MM-SL-SDR for the whole range of α and *N*, and for SNR ≤35 dB and $L\leq 10^{2}$.

### 6.2. Performance Analysis for the Source Localization

In this section, several energy-based acoustic source localization methods for the source localization without knowledge of the source transmission (or dilatation power), are compared with our proposed methods. Here, we include the weighted least-squares with one-step (labeled as WLS) in [24]. The SDP based source localization by jointly estimating the unknown source transmission (or radiation power) and the source location (labeled as ML-JE-Wang) is extended to the case with any energy decay factor, which is proposed by Wang in [27]. In addition, the extension of the Wang’s method under minimax criterion (labeled as MM-JE-Wang) is also compared in the simulation.

**Figure 8:**
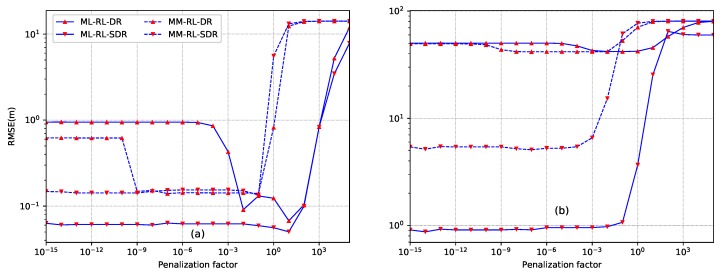
Comparison of the proposed source localization methods with different penalization factors. (**a**) the source is inside the convex hull of the sensor nodes ($y={\left[10,10\right]}^{T}$); (**b**) the source is outside the convex hull of the sensor nodes ($y={\left[10,80\right]}^{T}$).

*Example 5: selection of penalization factor.* Similarly with the case of sensor self-localization, the first example examines the sensitivity to the penalization factor. Figure 8 gives the results of RMSE versus penalization factors, where the source is located at $y={\left[10,10\right]}^{T}$ for Figure 8a and $y={\left[10,80\right]}^{T}$ for Figure 8b, respectively. N=12 sensors are located according to the setup (58). For these two scenarios, the source transmission (or radiation power) is S=1000, the SNR is set as SNR =40 dB, the number of sampling is L=1000, and the energy decay factor is α=1.8. It can be seen that the proposed ML based source localization methods are quite robust to the choice of penalization factors for both cases of inside and outside the convex hull. For the proposed SDR based ML-RL-SDR and MM-RL-SDR methods, any value in the range of $\left[10^{-15},10^{-2}\right]$ can be used without noticeable degradation of the localization performance for the case of inside convex hull, while any value in the range of $\left[10^{-15},10^{-3}\right]$ can be used for the case of outside convex hull. In comparison, the proposed direct relaxation based ML-RL-DR and MM-RL-DR methods, especially ML-RL-DR, are sensitive to the selection of penalization factors. The penalization factor for ML-RL-DR can be selected in the range of $\left[10^{-2},10^{1}\right]$ while that of MM-RL-DR can be selected in the range of $\left[10^{-9},10^{-1}\right]$ for the case of inside convex hull. In the following examples, we will select $\beta_{\mathrm{MLD}}=1$ and $\beta_{\mathrm{MLS}}=\beta_{\mathrm{MMD}}=\beta_{\mathrm{MMS}}=10^{-5}$ for the case of inside convex hull, and set all the penalization factors as $10^{-10}$ for the case of outside convex hull.

**Figure 9:**
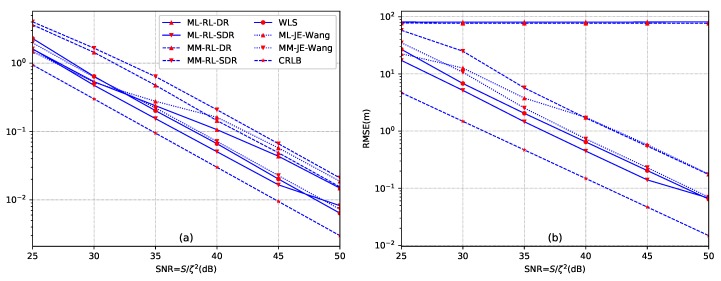
RMSE versus SNR. (**a**) the source is located at $y={\left[10,20\right]}^{T}$; (**b**) the source is located at $y={\left[100,30\right]}^{T}$.

*Example 6: performance analysis with different geometric layouts.* In this example, the performance comparison of several source localization methods including the proposed four methods, WLS, ML-JE-Wang and MM-JE-Wang, is given to reflect the impact of geometric layout of the sensors and the source. The CRLB by taking any energy decay factor into account is also included. The estimation results are given in Figure 9. N=12 sensors are fixed according to the setup (58). The source is located at $y={\left[10,20\right]}^{T}$ for Figure 9a and $y={\left[100,30\right]}^{T}$ for Figure 9b, which are inside and outside the convex hull formed by sensors, respectively. The energy decay factor is assumed to be known after iterative estimation, which is α=1.8. The sampling length for energy calculation is L=1000 and the transmission (or radiation power) S=1000 is unknown in prior. The number of Monte Carlo runs is $\bar{M}=1000$.

From Figure 9, we can clearly see that the performance of all the methods is close to each other in a wide range of SNR when the source is located inside the convex hull formed by the twelve sensors. In addition, all these methods can achieve very close performance to CRLB. In comparison, the proposed ML-RL-SDR method performs better than WLS, ML-JE-Wang, MM-JE-Wang over a wide range of SNR for both of these two scenarios. The performance of ML-RL-SDR exhibits degradation when the SNR is larger than 45 dB and is worse than MM-JE-Wang and WLS in these scenarios. Hence, we recommend the use of WLS and MM-JE-Wang for larger SNR. When the source is inside the convex hull, the proposed MM-RL-DR method provides better performance than MM-RL-SDR, and both of these two convex optimization based methods provide worse performance than ML-RL-DR. When the source is outside the convex hull formed by sensors, the direct norm relaxation based source localization methods (ML-RL-DR and MM-RL-DR) fail to give a correct estimate since the larger feasible region is formed due to the direct norm relaxation in such a scenario. By comparing Figure 9a,b, the overall performance of the scenario where the source is located outside the convex hull, is significantly poorer than that of the scenario where the source is located insider the convex hull.

**Figure 10:**
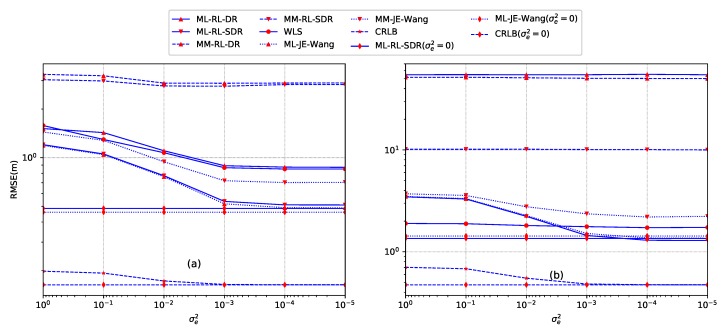
Comparison of source localization methods against energy decay factor error. (**a**) the source is located at $y={\left[20,30\right]}^{T}$; (**b**) the source is located at $y={\left[80,20\right]}^{T}$.

*Example 7: performance analysis against estimated error of the energy decay factor.* To evaluate the sensitivity to the error of energy decay factor, this example gives source localization performance of our proposed methods as well as the other methods. The energy decay factor is estimated according to (59), where Δα follows a truncated Gaussian distribution. The results against energy decay factor errors in terms of different variances for both the case of inside convex hull and the case of outside convex hull are given in Figure 10. The performance of ML-RL-SDR, ML-JE-Wang, and CRLB without estimation error ($\sigma_{e}^{2}=0$) is also included in the figure. The truth energy factor is α=2.2 and the truncated parameters are a=1.9 and b=2.5. N=12 sensors are deployed according to (58). For Figure 10a, the source is located at $y={\left[20,30\right]}^{T}$, SNR = 35 dB and the sampling size is L=500. For Figure 10b, the source is located at $y={\left[80,20\right]}^{T}$, SNR = 45 dB and the sampling size is L=1000. For both of the two cases, the source transmission power is set as S=1000.

From Figure 10a, we can see that the performance of the proposed ML-RL-SDR, ML-RL-DR methods, as well as the extended ML-JE-Wang, extended MM-JE-Wang and WLS improves with the variance of decay factor noise Δα increasing. ML-RL-SDR provides very close performance to ML-JE-Wang under a wide range of $\sigma_{e}^{2}$ and both of them provides better localization accuracy than the other methods. Furthermore, the performance of these two methods is approaching that of the case without estimate errors ($\sigma_{e}^{2}=0$). Two source localization methods under minimax criterion, MM-RL-DR and MM-RL-SDR, exhibit robust performance against errors of energy decay factor. When the source is outside the convex hull formed by sensors (see Figure 10b), the direct norm relaxation based source localization methods fail to accurately estimate the source location. The performance of MM-RL-DR and WLS does not change significantly with the variance $\sigma_{e}^{2}$ increasing. In addition, the proposed ML-RL-SDR method provides the close performance to the extended ML-JE-Wang method.

**Figure 11:**
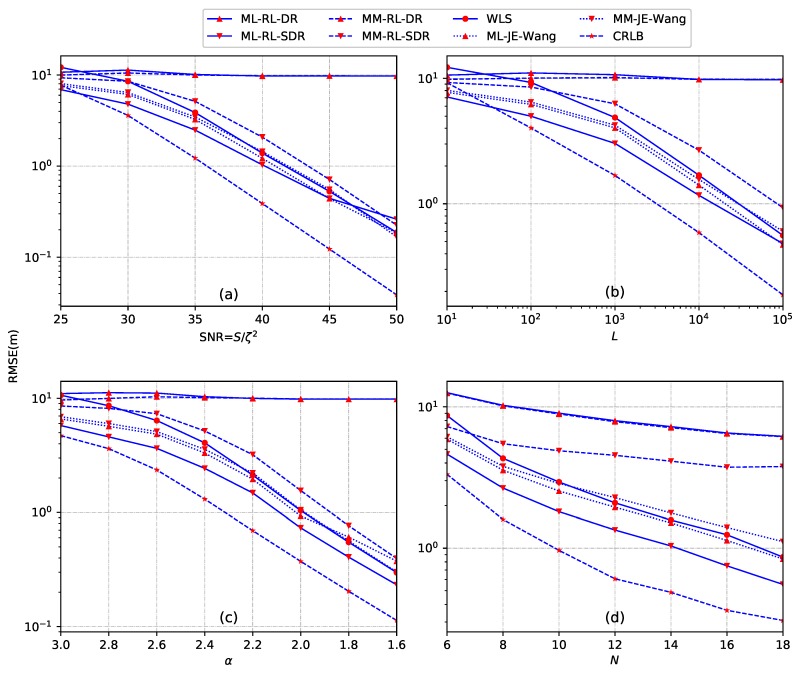
Performance analysis when the source is located randomly in a region of size 50 × 50 $m^{2}$. (**a**) RMSE versus SNR; (**b**) RMSE versus *L*; (**c**) RMSE versus α; (**d**) RMSE versus *N*.

*Example 8: sensitivity to the model parameters.* To illustrate how the parameters of energy decay model as well as the number of sensors impact the estimation performance, we performed $\bar{M}=5000$ Monte Carlo runs by using *N* uniformly distributed sensors in a region of size 50 × 50 $m^{2}$ to estimate the location of uniformly distributed source. Figure 11 gives performance versus source transmission (or radiation) SNR, sampling length *L*, energy decay factor α and number of sensors *N*, respectively, by averaging over all estimated source locations and noise realizations. The unknown transmission (or radiation) power S=1000. In Figure 11a, we set L=500, α=2.4 and N=8. In Figure 11b, we set SNR =35 dB, α=2.6 and N=8. In Figure 11c, we set SNR =35 dB, L=500 and N=8. In Figure 11d, we set SNR =30 dB, L=1000 and α=2.2.

From Figure 11, it can be obviously seen that the proposed ML-RL-SDR provides the best performance in a wide range of SNR, *L*, α and *N*. However, there is an exception. The performance of ML-RL-SDR exhibits some performance degradation when SNR ≥50 dB. The extended ML-JE-Wang is shown to be better than MM-JE-Wang for the entire range of all the parameters. The proposed MM-RL-SDR method performs worse than ML-based source localization methods and WLS with SNR, *L*, α and *N* increasing. Similarly with the previous examples, for the direct norm relaxation based methods (ML-RL-DR and MM-RL-DR), they fail to give the accurate estimate since the impact is averaged over all potential topologies of sensors and source. In addition, the WLS method shows significant performance degradation for small SNR (SNR ≤30 dB), small sampling number *L* ($L\leq 10^{2}$), large decay factor α (α≥2.8) and a small number of sensors *N* (N≤8). By comparing the ML-based and MM-based source localization methods, ML-based methods generate better performance than MM-based methods for the whole range of all the four parameters. This is because the ML-based methods utilize the distribution of the energy noise, which is not considered in the MM-based methods. That is, the ML-based methods utilize more information than the MM-based methods in this example. Note that a significant gap exists between the CRLB and all the mentioned methods. This observation illustrates that there still exists room for the performance improvement of the acoustic energy based methods. In addition, the performance of MM-RL-SDR does not improve significantly when the number of the sensors is increasing.

*Complexional computation:* To illustrate the computational cost of our proposed source localization algorithms, we give a comparison of the average CPU time in Table 2. The extended WLS in [24] and the Wang’s ML-JE-Wang in [27] are also included in the comparison. The results are also derived by using an Intel Xeon core E5-1630 PC with 3.7 GHz CPU and 16 G RAM. We can see that WLS requires the least time in all the algorithms. The proposed ML-RL-SDR, MM-RL-SDR as well as ML-JE-Wang algorithms exhibit almost same estimation time, which have less time than ML-RL-DR and MM-RL-DR algorithms.

*Simulation summary:* When the source transmission power (or radiation power) *S* is unknown, we propose two kinds of source localization methods using direct norm relaxation and SDR respectively under ML and minimax criterion, respectively. From above simulations, we have the following conclusions:

- The proposed SDR-based source localization methods, especially ML-RL-SDR, provide a comparable source location estimate for both the case of inside convex hull formed by sensors and the case of outside convex hull. In comparison, the proposed direct norm relaxation based source methods failed to provide accurate source location estimate when the source is located outside the convex hull.
- The proposed SDR-based source localization methods, as well as extended ML-JE-Wang and MM-JE-Wang provide a robust estimate against errors of the energy decay factor noise.
- When the sensors and the source are randomly and uniformly distributed in a square region, the proposed ML-RL-SDR provides superior performance in a wide range of SNR, sampling number *L*, energy decay factor α and number of sensors *N*.

## 7. Conclusions

In this paper, a set of sensor self-localization and source localization methods based on the acoustic energy decay model are developed and compared. For the application of sensor self-localization, a so-called direct norm relaxation and semidefinite relaxation are utilized to generate two convex optimization based localization methods. The original optimization problems are converted into convex ones, which is reliably and efficiently solved by the Interior-point methods. For the application of the source localization without knowledge of the transmission power, we derived the ML and minimax optimization formulations based on the transmission power elimination with all pairwise acoustic energy readings. Again, two kinds of source localization methods by utilizing direct norm relaxation and SDR are derived. In addition, the CRLB with any energy decay factor for both the sensor self-localization with known transmission power and the source localization without knowledge of the transmission power is derived. Simulation results demonstrate that the proposed methods can provide comparable or even better performance than the existing methods.

It is worth noting that the proposed methods consider the estimation without the communication errors between the sensors and the fusion center, which can deteriorate the estimation performance. The source localization method by taking the communication errors into account will be studied in the future work.

## Figures and Tables

**Table 1:** Average CPU estimation time for different source localization algorithms for sensor self-localization.

Algorithm	Average CPU Estimation Time (ms)
ML-SL-DR	404.2
ML-SL-SDR	200.6
MM-SL-DR	340.4
MM-SL-SDR	197.6
ML-Searching-T	4.8
ML-Searching-F	8.2

**Table 2:** Average CPU estimation time for different source localization algorithms for source localization.

Algorithm	Average CPU Estimation Time (ms)
ML-RL-DR	631.9
ML-RL-SDR	235.6
MM-RL-DR	368.8
MM-RL-SDR	243.9
WLS	6.8
ML-JE-Wang	245.0
MM-JE-Wang	295.9

## References

[1] Srbinovska M., Gavrovski C., Dimcev V., Krkoleva A., Borozan V. (2015). Environmental parameters monitoring in precision agriculture using wireless sensor networks. J. Clean. Prod..

[2] Wu M., Tan L., Xiong N. (2016). Data prediction, compression, and recovery in clustered wireless sensor networks for environmental monitoring applications. Inf. Sci..

[3] Du R., Chen C., Yang B., Lu N., Guan X., Shen X. (2015). Effective urban traffic monitoring by vehicular sensor networks. IEEE Trans. Veh. Technol..

[4] Kumar N., Misra S., Obaidat M.S. (2015). Collaborative learning automata-based routing for rescue operations in dense urban regions using vehicular sensor networks. IEEE Syst. J..

[5] Sheng X., Hu Y.H. (2005). Maximum likelihood multiple-source localization using acoustic energy measurements with wireless sensor networks. IEEE Trans. Signal Process..

[6] Yan Y., Wang H., Shen X., Zhong X. (2015). Decision Fusion with Channel Errors in Distributed Decode-Then-Fuse Sensor Networks. Sensors.

[7] Yan Y., Wang H., Xue T., Wang X. (2017). Optimal local sensor decision rule for target detection with channel fading statistics in multi-sensor networks. J. Frankl. Instit..

[8] Zou T., Li Z., Li S., Lin S. (2017). Adaptive Energy-Efficient Target Detection Based on Mobile Wireless Sensor Networks. Sensors.

[9] Yu Y. (2017). Distributed target tracking in wireless sensor networks with data association uncertainty. IEEE Commun. Lett..

[10] Pak J.M., Ahn C.K., Shi P., Shmaliy Y.S., Lim M.T. (2017). Distributed hybrid particle/FIR filtering for mitigating NLOS effects in TOA-based localization using wireless sensor networks. IEEE Trans. Ind. Electron..

[11] Gao S., Zhang F., Wang G. (2017). NLOS Error Mitigation for TOA-Based Source Localization with Unknown Transmission Time. IEEE Sens. J..

[12] Xu E., Ding Z., Dasgupta S. (2011). Source localization in wireless sensor networks from signal time-of-arrival measurements. IEEE Trans. Signal Process..

[13] Shen H., Ding Z., Dasgupta S., Zhao C. (2014). Multiple Source Localization in Wireless Sensor Networks Based on Time of Arrival Measurement. IEEE Trans. Signal Process..

[14] Xu E., Ding Z., Dasgupta S. (2011). Reduced complexity semidefinite relaxation algorithms for source localization based on time difference of arrival. IEEE Trans. Mob. Comput..

[15] Huang B., Xie L., Yang Z. (2015). TDOA-based source localization with distance-dependent noises. IEEE Trans. Wirel. Commun..

[16] Zou Y., Liu H., Xie W., Wan Q. (2017). Semidefinite Programming Methods for Alleviating Sensor Position Error in TDOA Localization. IEEE Access.

[17] Ho K. (2012). Bias reduction for an explicit solution of source localization using TDOA. IEEE Trans. Signal Process..

[18] Yan Q., Chen J., Ottoy G., Cox B., De Strycker L. An accurate AOA localization method based on unreliable sensor detection. Proceedings of the 2018 IEEE Sensors Applications Symposium (SAS).

[19] So H.C., Lin L. (2011). Linear Least Squares Approach for Accurate Received Signal Strength Based Source Localization. IEEE Trans. Signal Process..

[20] Tomic S., Beko M., Dinis R. (2015). RSS-based localization in wireless sensor networks using convex relaxation: Noncooperative and cooperative schemes. IEEE Trans. Veh. Technol..

[21] Ouyang R.W., Wong A.S., Lea C.T. (2010). Received signal strength-based wireless localization via semidefinite programming: Noncooperative and cooperative schemes. IEEE Trans. Veh. Technol..

[22] Li D., Hu Y.H. (2003). Energy-based collaborative source localization using acoustic microsensor array. EURASIP J. Appl. Signal Process..

[23] Meng W., Xiao W., Xie L. (2011). An efficient EM algorithm for energy-based multisource localization in wireless sensor networks. IEEE Trans. Instrum. Meas..

[24] Meesookho C., Mitra U., Narayanan S. (2008). On energy-based acoustic source localization for sensor networks. IEEE Trans. Signal Process..

[25] Yan Y., Wang H., Wang X. A novel least-squares method of source localization based on acoustic energy measurements for UWSN. Proceedings of the 2011 IEEE International Conference on Signal Processing, Communications and Computing (ICSPCC).

[26] Yan Y., Wang H., Shen X., Yang F., Chen Z. Efficient convex optimization method for underwater passive source localization based on RSS with WSN. Proceedings of the 2012 IEEE International Conference on Signal Processing, Communication and Computing (ICSPCC 2012).

[27] Wang G. (2011). A semidefinite relaxation method for energy-based source localization in sensor networks. IEEE Trans. Veh. Technol..

[28] Wang G., Li Y., Wang R. (2013). New Semidefinite Relaxation Method for Acoustic Energy-Based Source Localization. IEEE Sens. J..

[29] Yuan J., Ai W., Deng H., Shuai T., Zhao X. (2015). Exact solution of an approximate weighted least squares estimate of energy-based source localization in sensor networks. IEEE Trans. Veh. Technol..

[30] Lohrasbipeydeh H., Gulliver A., Amindavar H. (2014). A minimax SDP method for energy based source localization with unknown transmit power. IEEE Wirel. Commun. Lett..

[31] Meng C., Ding Z., Dasgupta S. (2008). A semidefinite programming approach to source localization in wireless sensor networks. IEEE Signal Process. Lett..

[32] Cheng C., Hu W., Tay W.P. Localization of a moving non-cooperative RF target in NLOS environment using RSS and AOA measurements. Proceedings of the 2015 IEEE International Conference on Acoustics, Speech and Signal Processing (ICASSP).

[33] Tomic S., Beko M., Dinis R., Montezuma P. (2017). Distributed algorithm for target localization in wireless sensor networks using RSS and AoA measurements. Pervasive Mob. Comput..

[34] Sivrikaya F., Yener B. (2004). Time synchronization in sensor networks: a survey. IEEE Netw..

[35] Luo Z.Q., Ma W.K., So A.C., Ye Y., Zhang S. (2010). Semidefinite relaxation of quadratic optimization problems. IEEE Signal Process. Mag..

[36] Boyd S., Vandenberghe L. (2004). Convex Optimization.

[37] Ozdemir O., Niu R., Varshney P.K. (2009). Channel aware target localization with quantized data in wireless sensor networks. IEEE Trans. Signal Process..

[38] Sundaresan A., Varshney P.K. (2011). Location estimation of a random signal source based on correlated sensor observations. IEEE Trans. Signal Process..

[39] Luo Z., Jannett T.C. Energy-based target localization in multi-hop wireless sensor networks. Proceedings of the 2012 IEEE Radio and Wireless Symposium.

[40] Yang X., Niu R., Masazade E., Varshney P.K. (2013). Channel-aware tracking in multi-hop wireless sensor networks with quantized measurements. IEEE Trans. Aerosp. Electron. Syst..

[41] Sturm J.F. (1999). Using SeDuMi 1.02, a MATLAB toolbox for optimization over symmetric cones. Optim. Methods Softw..

[42] Toh K.C., Todd M.J., Tütüncü R.H. (2012). On the implementation and usage of SDPT3–a Matlab software package for semidefinite-quadratic-linear programming, version 4.0. Handbook on Semidefinite, Conic and Polynomial Optimization.

[43] Grant M., Boyd S. (2014). CVX: Matlab Software for Disciplined Convex Programming, Version 2.1 (2014). http://cvxr.com/cvx.

[44] Lagarias J.C., Reeds J.A., Wright M.H., Wright P.E. (1998). Convergence properties of the Nelder–Mead simplex method in low dimensions. SIAM J. Optim..

